# The downward spiral of mental disorders and educational attainment: a systematic review on early school leaving

**DOI:** 10.1186/s12888-014-0237-4

**Published:** 2014-08-27

**Authors:** Pascale Esch, Valéry Bocquet, Charles Pull, Sophie Couffignal, Torsten Lehnert, Marc Graas, Laurence Fond-Harmant, Marc Ansseau

**Affiliations:** Centre for Health Studies, Centre de Recherche Public de la Santé, Rue Thomas Edison 1 A-B, Strassen, 1445 Luxembourg; Department of Clinical Sciences, University of Liège, Avenue de l’Hôpital 13, Liège, 4000 Belgium; Competence Centre of Methodology and Statistics, Centre de Recherche Public de la Santé, Rue Thomas Edison 1 A-B, Strassen, 1445 Luxembourg; Department of Psychiatry, Centre Hospitalier de Luxembourg, Rue Ernest Barblé 4, Luxembourg, 1210 Luxembourg; Department of Psychiatry, Centre Hospitalier du Kirchberg, Rue Edward Steichen 9, Luxembourg, 2540 Luxembourg; Centre Hospitalier Neuro-Psychiatrique, Avenue des Alliés 17, Ettelbruck, 9002 Luxembourg

## Abstract

**Background:**

Most psychiatric disorders present symptom patterns that cause severe impairment on the emotional, cognitive and social level. Thus, adolescents who suffer from a mental disorder risk finding themselves in a downward spiral caused by the reciprocal association of psychological symptoms and negative school experiences that may culminate in early school leaving. In addition to previous collective work that mainly focused on school refusing behaviour among children and was presented as an expert’s opinion, the following systematic review fills the knowledge gap by providing a structured overview of the bidirectional association between mental health and secondary school dropout based on a sound methodology and with a particular focus on mediating factors.

**Methods:**

Four electronic databases were searched from January 1990 until June 2014. Selected references were assessed for study details, main results, mediating factors and methodological limitations. Standardized risk of bias assessment was conducted.

**Results:**

Mood and anxiety disorders seemed to have a less consequential direct effect on early school leaving than substance use and disruptive behaviour disorders. The association between externalizing disorders and educational attainment was even stronger when the disorder occurred early in life. On the other hand, internalizing disorders were reported to develop as a consequence of school dropout. Only few studies had addressed gender differences, with discrepant results. Socio-economic background, academic achievement and family support were identified as significant mediating factors of the association between mental disorders and subsequent educational attainment.

**Conclusions:**

Findings suggested a strong association between mental health and education, in both directions. However, most studies focused on mediating factors that could not be targeted by intervention programs.

**Electronic supplementary material:**

The online version of this article (doi:10.1186/s12888-014-0237-4) contains supplementary material, which is available to authorized users.

## Background

According to Berg, problems of school attendance first gained clinical consideration in 1913 in C.G. Jung’s work on child neurosis [1]. However, early dropout research primarily focused on demographic and scholastic risk factors related to educational attainment. An extensive synthesis is provided in the early work of Rumberger [2]. An opening in addressing psychological characteristics of early school leavers had been presented in a set of guidelines published by Schreiber in 1965 [3]. Later, in 1972, Bachman found out that male high school dropouts, as compared to students who stayed in school, showed more delinquent behaviour, lower self-esteem and feelings of self-efficacy; and that these characteristics were evident prior to their dropping out [4].

A major contribution to dropout research in terms of mental health had been done by Kessler who evaluated the negative effects of early onset psychiatric disorders on educational attainment [5]. Most psychiatric disorders present symptom patterns that cause severe impairment on the emotional, cognitive and social level, inasmuch as the student affected by a disorder may be unable to carry out his/her academic or vocational potential. Thus, adolescents who suffer from a mental disorder risk finding themselves in a downward spiral caused by the interrelation of psychological symptoms and negative school experiences that may culminate in early school leaving. In turn, leaving school without any qualifications may implicate a difficult professional and social integration regarding limited educational and vocational opportunities, thus involving an increased vulnerability for developing a mental disorder.

A mechanism underlying the reciprocal association between psychological characteristics and educational attainment had been proposed by Roeser who claimed that the perception of an adverse scholastic event was influenced by individual coping attitudes that would define whether negative feelings were internalized or externalized [6]. If referring to an internalizing coping style, the student tended to direct negative emotions against him-/herself, whereas adolescents who tended to externalize negative feelings, turned their anger or frustration against others. The latter may represent the stereotypical “troublemaker” in class, a male student who accumulates academic failure, behavioural problems and subsequent disciplinary measures before finally dropping out of school. Previous collective work regarding the association between mental health and education mostly focused on absenteeism among children and younger adolescents and had reached a consensual differentiation between school refusing behaviour that would be related to internalizing symptoms and truancy that would co-occur with externalizing disruptive behaviour [1,7-9]. However, those early literature reviews emphasized the heterogeneous process of early school leaving and the need to consider the interplay of multiple factors. Indeed studies focusing on the association between mental disorders and school dropout should control for the mediating effect of socioeconomic factors [10,11], family characteristics [12,13] and school-related experiences [14,15], in order to provide preferably unbiased results that may guide the conception of policies and intervention programs. Indeed, both levels of action often share the same weaknesses as programs mainly rely on educational or academic features and thus may not be sensitive enough to detect early school leavers who refer to an internalizing coping style [16]. In contrast to the so-called troublemakers, those students who silently initiate a process of alienation are often underdiagnosed and thus ignored by intervention programs [16]. Furthermore, to our knowledge, previous review work has either been presented as an expert’s opinion paper providing a more general synthesis of the problem or, when referring to a systematic methodology, the review only tackled one specific disorder category [17]. Therefore, in addition to previous collective work, the present systematic review aims to fill the knowledge gap in several ways. First, in an attempt to complement work focusing on school refusing behaviour among children or on dropout from higher education [18], the following systematic review concentrates on dropping out of secondary education as it involves the end of compulsory schooling but also the period of life where up to 50% of chronic mental disorders have their onset [19]. Second, in addition to the more general character of cited opinion papers, it provides a systematic structured overview of the topic, based on a sound methodology including pre-defined criteria for the selection and analysis of retrieved references and a standardized risk of bias assessment to evaluate their quality. Finally, the review will not be limited to a specific disorder category but tends to identify which type of mental disorder seems to be most strongly related to educational attainment. More precisely, summarizing existing knowledge about the reciprocal association between mental health and early school leaving will be guided by the following questions:

– What types of psychiatric disorders seem to be the most impeding for educational attainment (school dropout as the outcome)?

– What types of psychiatric disorders seem to develop after dropping out of school (school dropout as a predictor)?

– Are gender differences being addressed?

– What are important confounding factors of the mental health - dropout association that may guide the conception of intervention programs?

– According to a standardized risk of bias assessment, what are the main methodological concerns that may guide the conception of future research?

## Methods

### Search strategy

Relevant computerized databases were searched for peer-reviewed articles published between January 1990 and June 2014. The start date was set at 1990 to target references referring to diagnostic criteria not earlier than the Diagnostic and Statistical Manual of Mental Disorders, third version (DSM III), as this version implied a major shift from a psychodynamic perspective of disorder in favour of an empirical foundation [20]. Databases included Medline, Embase, PsycINFO, and ERIC (Education Resources Information Center). The search design was developed with a librarian. Search terms comprised key words related to “adolescent” (student, young adult, adolescent), “education” (school, educational status, student dropouts) and “mental health” (mental disorders) used in different combinations and adapted to the query language of the database. Details are available upon request.

### Eligibility criteria

References were considered eligible for analysis if they matched the following criteria:

– References had to be published in a scientific peer-reviewed journal and present an empirical investigation of the association between psychological disorders and school dropout. We differentiated between studies considering school dropout as a predictor of mental health problems and studies considering school dropout as the outcome of interest.

– Considered studies should refer to a cross-sectional, case–control or longitudinal design. Meta-analyses were also accepted.

– To broaden coverage, references in English, French and German were considered.

– School dropout had to occur during secondary education (e.g. high school) and refer to having left school prior to graduation. Selected references had to indicate that the period of reference was secondary education.

– To induce some homogeneity in the methods regarding the assessment of psychiatric disorders, except for substance use, diagnostic tools had to refer to the criteria of the “American Psychiatric Association” (DSM IV-R, DSM IV, DSM III-R or DSM III depending on the period of reference) as they are considered the reference standard [21].

– According to a review on trends in adolescent illicit substance use [22], patterns of use were very heterogeneous. However, experimentation patterns were more common in young ages than abuse or dependence disorders. Thus, considering only references that referred to the diagnostic criteria of DSM would be too restrictive and omit relevant information on problematic substance use. Therefore, we decided to include references that evaluated substance use by reported frequencies. Definitions had to be indicated in the paper.

### Data analysis

As references were considered significantly heterogeneous in terms of study design, characteristics of study population and considered confounding, a comprehensive descriptive analysis was preferred to an attempt of pooling extracted data.

Extracted information of included references is presented in separate Additional files 1, 2, 3 and targets a description of the study design, the characteristics of the study population, considered psychiatric disorders and the tools to evaluate them, included confounding factors, main adjusted results illustrated by mean differences, adjusted odds ratios or relative risks as well as an overall estimation of the quality of the study. Therefore, observational studies were evaluated with the revised RTI Item Bank developed by Viswanathan et al. [23] whereas meta-analyses were rated according to guidelines of “Meta-analysis Of Observational Studies in Epidemiology” (MOOSE) [24].

The RTI Item Bank provides 16 validated items to assess different types of bias. The authors guide the user in considering and evaluating the items according to specific study designs. For the scope of this review, items related to selection, detection, attrition and reporting bias were applied and combined to provide an overall estimation of risk of bias. A study with one or more key domains estimated as unclear or negative, could not be labelled as of low risk of bias. As the present review aims to provide a comprehensive overview of published work, no studies were excluded a priori from the results section, however, the reader was cautioned when results were extracted from studies with an unclear or high risk of bias and the “discussion” part includes a comparison of major results in terms of attributed risk of bias per disorder category.

Confounding was evaluated according to identified major domains including socio-demographic, family and school-related characteristics. Studies controlling for at least two of the depicted domains were considered to have minimized their risk of bias related to confounding. However, with regard to the heterogeneity of study objectives and hypotheses, confounding was not included into the overall risk of bias estimation.

## Results

**Figure 1:**
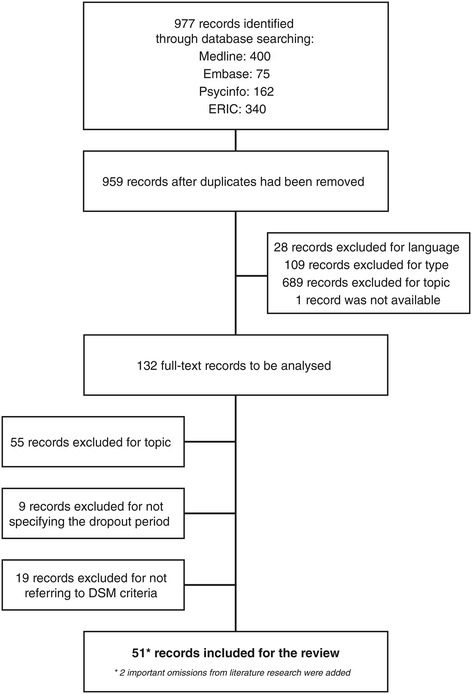
**Selection of records, adapted from the PRISMA statement, 2009.**

As the search terms were chosen to be broad-spectrum, the initial search from the 4 databases resulted in a total of 959 references after duplicates had been removed. A first exclusion of 28 records was based on language and another 109 records were excluded as they did not present original data (e.g. descriptive reviews, case reports, opinion papers). After screening the title and the abstract, 689 references were excluded as they did not investigate the relationship between mental disorders and school dropout and 1 reference was not available. Finally, 132 full-text records were assessed for eligibility. Fifty-five were excluded for not matching the topic, 9 did not specify the period of school dropout and 19 did not refer to diagnostic criteria according to the DSM. The flowchart below, adapted from the “Preferred Reporting Items for Systematic Reviews and Meta-Analyses” (PRISMA) statement [25], illustrates the progressive screening and inclusion of relevant records for the review. Included references were supplemented by two important omissions from electronic literature research [10,15] Figure 1.

Most of the included references (33 out of 51) were published in the last 10 years, indicating an increasing concern for the association between school dropout and mental disorders. Only 8 studies had been conducted outside Anglophone countries [26-33].

Regarding the direction of the association, 29 references considered school dropout as the outcome, whereas 17 considered it a predictor of mental health problems. Five records investigated both directions of this association. The synthesis of all included references is provided as Additional files 1, 2 and 3.

### Methodological issues and risk of bias assessment

The large majority of references investigating psychiatric disorders as predictors of high school dropout were longitudinal (24 out of 34), with 2 references providing an additional case control design [34,35]. Another 9 references presented a cross-sectional design and one was a meta-analysis [36]. Considering the distribution of longitudinal versus transversal studies, the urge of earlier reviews claiming for prospective follow-up studies in dropout research had been appreciated [2]. However, although a longitudinal study design is considered the gold standard to draw the trajectory of meaningful events and to allow for a causal interpretation of their association with school completion or dropout, conclusions should be taken with caution [37].

**Figure 2:**
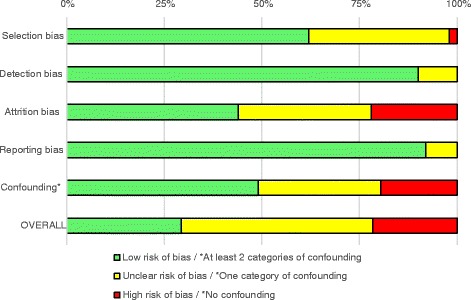
**Summary of risk of bias assessment.** *Green: At least 2 types of confounding variables, among sociodemographic, family and academic variables. Yellow: One type of confounding variables. Red: 0 type of confounding variables.

Indeed, regarding standardized risk of bias assessment, only 29% of included studies were estimated as having a low risk of bias whereas 22% were rated as of high risk. The remaining studies did not provide enough detailed information for at least one of the key biases assessed and were thus rated as of unclear risk (Figure 2 and Additional files 4 and 5). In most studies with an overall risk of bias rated as high or unclear, it was difficult to evaluate whether the studied sample was representative of the target population (selection bias) or whether results were biased by differential nonresponse or loss to follow-up (attrition bias).

Another weakness in the methods was the use of retrospective self-reported data regarding childhood adversities [29,38], psychiatric disorders [5,39-41] and substance use [42-47] which may induce a recall bias. However, considering the difficulties to reach a target population of early school leavers, the use of retrospective data seemed appropriate. Only one study controlled for the accuracy of self-reported nicotine use by analysing a saliva sample [48].

### Mental disorders as a predictor of school dropout

#### Substance use

Almost half of the included records focused on substance use as a predictor of secondary school dropout. The age of onset and different patterns of use were investigated for their impact on educational attainment.

The deleterious effect of cannabis use on educational attainment seemed to be even stronger when occurring early in life [26,49,50]. Indeed, school dropout was more common when cannabis use was reported before the 9th grade, with adjusted odds ratios varying from 2.05 (95% CI: 1.41 to 2.99) for boys to 3.41 (95% CI: 1.89 to 6.13) for girls [26]. In their meta-analysis, Horwood et al. came to a similar conclusion [36]. They estimated that the early use of cannabis accounted for 17% of the overall rate of high school dropout with statistical adjustment made for socio-demographic characteristics, child cognitive ability and family functioning. Only one study identified an association similar in magnitude for ages 16, 17 and 18 with a corresponding odds ratio of 2.31 (95% CI: 1.49 to 3.60) [51]. However, their results may be biased by a significant loss to follow-up.

In 2003, Fergusson investigated the frequency of cannabis use and reported that adolescents who had used cannabis on more than 100 times by the age of 16, were 3.7 times more likely to drop out of high school than those who had never used cannabis (95% CI: 1.8 to 7.5, p < 0.001) [52]. Later, in 2009, Marti analysed different patterns of cannabis use among girls and found out that moderate escalating use was the strongest predictor of high school dropout (OR: 5.56, p < 0.01), followed by problematic use (OR: 5.33, p < 0.001) and late-heavy use (OR: 4.45, p < 0.01) [53]. However, results may be biased by a considerable loss to follow-up (44%) and the omission of potentially confounding factors. Indeed, the results of another study which seemed to confirm higher odds of high school dropout for cannabis users compared to non-users, had become insignificant after introducing nicotine consumption into the model [54].

An unexpected result was reported by Legleye who found that adolescents who only experimented with the use of cannabis without changing to subsequent daily use were less likely to drop out of secondary school than adolescents who never used cannabis (OR adjusted: 0.80; 95% CI: 0.64 to 1.00 for boys and 0.64; 95% CI: 0.48 to 0.85 for girls) [26].

Compared to total abstinence, substance use disorders (abuse or dependence) can be seen as the other extreme on a continuum of use. Odds ratios expressing the magnitude of their association with high school dropout varied from 1.34 (95% CI: 1.11 to 1.62 [39]), to 2.3 (95% CI: 1.8 to 3.1; p < 0.05 [5]), 2.48 (95% CI: 1.30 to 4.74 [38]) and 2.9 (95% CI: 2.1 to 4.0; p < 0.00 [40]) with statistical controls made for childhood adversities, socio-demographic characteristics and prior mental disorders. Surprisingly, Breslau did not identify an additional risk for school dropout when considering the progression from use to use disorders (abuse or dependence) [39].

Only one study investigated another illicit drug than cannabis and focused on amphetamine. As for cannabis, early-onset amphetamine users had higher odds for school dropout than non-users (OR adjusted for gender, parental smoking and divorce: 2.7; 95% CI: 1.8 to 3.9; p < 0.01). But when additionally adjusted for cannabis use, the odds decreased to insignificance [55].

Moreover, not only illicit substance use was supposed to have an impact on educational attainment, but also the consumption of nicotine [26,48] and alcohol [39]. Indeed, Legleye [26] identified an association between smoking at the age of 17 and subsequent school dropout, with odds ratios of 2.62 for boys (95% CI: 2.15 to 3.20) and 2.92 for girls (95% CI: 2.28 to 3.74), adjusted for socio-demographic characteristics and grade repetition. This result was confirmed by Breslau [39] who detected a weaker but still significant association between nicotine dependence and high school dropout (OR adjusted for socio-demographic characteristics and childhood adversities: 1.52; 95% CI: 1.13 to 2.03). However, none of the included studies detected a significant association between alcohol consumption and high school dropout. Furthermore, as for cannabis, alcohol experimentation only, as compared to total abstinence, seemed to have a positive effect on educational attainment [26].

### Internalizing disorders

Internalizing disorders refer to anxiety and mood disorders, both associated with educational attainment [5,32,33,40,56-60].

When adjusted for socio-demographic factors, mood disorders were significantly related to school dropout with varying odds ratios decreasing from 3.38 for major depression [58], to 2.75 (95% CI: 1.18 to 6.42, p < 0.01) for the early onset of depressive symptoms [32], and down to 1.4 (95% CI: 1.2 to 1.6, p < 0.01) for any mood disorder [56]. Another strong predictor of early school leaving and often co-occurring with mood disorders were suicidal ideations, with an odds ratio of 7.29 adjusted for socio-demographic characteristics and psychiatric morbidity [58].

Among anxiety disorders, after controlling for potentially confounding factors, social phobia was a strong predictor of poor educational outcomes (OR adjusted: 0.17; 95% CI: 0.04 to 0.70 [28]). Thereby, McShane confirmed the work of Van Ameringen [29] who reported that the main reasons for dropping out of school, as indicated by early school leavers themselves, were feeling too nervous in class and being anxious to speak in public, both representing symptoms of social phobia [60]. However, as both studies were conducted on a small clinical sample with differential loss to follow-up or lacking information about the response rate, results should be considered with caution. Odds ratios expressing the impact of any anxiety disorder on high school dropout varied from 1.3 (95% CI: 1.2 to 1.4; p < 0.01 [56]) to 1.4 (95% CI: 1.1 to 1.8, p < 0.05 [5]), with one study even reporting reduced odds for high school dropout among young people with a generalized anxiety or post-traumatic stress disorder [29].

### Externalizing disorders

Externalizing disorders refer to disruptive behaviour disorders including conduct disorder, oppositional defiant disorder and antisocial personality, and thus representing the stereotypical behaviour of a school dropout who acts out his/her frustration. Indeed, disruptive behaviour disorders including early childhood conduct disorders in particular, had an important negative effect on educational attainment. Based on the National Comorbidity Survey in the US, Kessler detected that eighth grade graduates presenting a conduct disorder were 2.4 times more likely to drop out of high school than their peers without any disorder (95% CI: 1.8 to 3.2; p < 0.05) [5]. Statistical controls had been made for socio-demographic correlates. Kessler’s results were confirmed by several subsequent studies that reported adjusted odds ratios varying from 1.35 (95% CI: 1.08 to 1.69 [31]) to 1.89 (95% CI: 1.57 to 2.26 [39]), 2.38 (95% CI: 1.43-3.96 [38]) 3.35 [10] and up to 6.74 [58].

Odds ratios expressing the magnitude of the association between any impulse control disorder and subsequent school dropout decreased from 2.2 (95% CI: 1.8 to 2.7, p < 0.01) in high-income countries to 1.3 (95% CI: 1.1 to 1.6, p = 0.01) in low-income countries suggesting a moderating effect of socio-economic characteristics [56].

### Other mental disorders

Several studies investigated the association between attention deficit disorder with or without hyperactivity (ADHD) and early school leaving [10,34,35,39,61]. In 1999, Hansen noted a difference between ADHD adolescents and controls in graduating from high school (*χ*2 = 3.46, p = 0.63), but no significant difference between the 2 groups in subsequent occupational status [61]. However, these observations should be interpreted with caution as they were drawn from a very small sample after a significant loss to follow-up.

When adjusting for gender and age, adolescents with ADHD were 2.7 times more likely to drop out of high school than their peers without any disorders (95% CI: 1.8 to 3.9, p < 0.001) [34]. Breslau investigated all 3 types of ADHD separately and reported that the association was strongest for the combined type (OR: 2.06; 95% CI: 1.66 to 2.56). Additional control was made for socio-demographic characteristics, childhood adversities and co-occurring mental disorders [39]. However, when controlling for the potentially mediating effect of IQ and reading ability, the negative effects of attention deficit disorder on educational attainment lost significance [10].

In a clinical sample of adolescents hospitalized for a first episode of non-affective psychosis, 44% had dropped out of high school which was a much higher rate than the reported high school dropout rates of 12.8 to 17.8% from the counties where the study was conducted [62]. The most common diagnosis for early school leavers were schizophrenia, paranoid type (45.8%). Furthermore, high school dropouts were significantly younger at the time of hospitalization, confirming the deleterious effect of early onset psychiatric disorders on educational attainment. However, as key items for risk of bias assessment were rated unclear because of lacking information regarding representativeness and response rate, the reported results had to be considered with caution.

### School dropout as a predictor of mental health problems

#### Substance use

When considering school dropout as a predictor, the focus of retrieved records regarding mental health consequences was very consistent: 14 out of 22 studies investigated the effect of early school leaving on substance use.

Results regarding subsequent cannabis use were heterogeneous. High school dropout seemed to increase the probability of later cannabis use for both, boys and girls [28,30,63-65].

However, after controlling for a consistent number of potentially confounding factors, Fergusson [52] and D’Amico [42] did not detect such a significant association.

Obot conducted 3 case control studies to investigate the association between school dropout and injecting drug use [45-47]. Among Non-Hispanic White Americans and African Americans, early school leavers without the high school equivalency credential “General Educational Development” (GED) would be more likely to have started injecting drugs (OR adjusted for age and gender: 2.2; 95% CI: 1.31 to 3.60, p = 0.003 and OR adjusted: 1.9; 95% CI: 1.3 to 2.6, p < 0.001 respectively) and this association would become even stronger for dropouts with a GED (OR adjusted: 3.4; 95% CI: 1.99 to 5.71, p < 0.001 and OR adjusted: 2.3; 95% CI: 1.4 to 3.8, p < 0.001 respectively). The same results had been reported for recent injecting drug use. Among White American youth, only dropouts with a GED would be more likely to be recent drug injectors (OR adjusted: 4.4; 95% CI: 1.31 to 14.96, p = 0.017) whereas, among African American youth, both dropouts with and without a GED would be more likely to be recent drug injectors than graduates. However, the reported studies lacked information about the response rate and the selection of the comparison group, resulting in an overall risk of bias rated as unclear. Furthermore, results had only been adjusted for socio-demographic characteristics.

Two recent studies reported that high school dropouts were more at risk to develop a substance use disorder (OR adjusted for socio-demographic characteristics and service use: 2.16; 95% CI: 1.32 to 3.52; p < 0.05 [27] and OR adjusted for scholastic characteristics: 3.5; 95% CI: 1.71 to 7.17; p < 0.001 [43]). Here, this risk was reduced by obtaining a GED or training after dropout.

When compared to in-school youth, dropouts were also more likely to be current smokers (OR adjusted for age, gender and ethnicity: 2.59; 95% CI: 2.17 to 3.08, p < 0.01 [66] and OR adjusted for problem behaviour: 1.6 [67]).

As for cannabis, the association between educational attainment and subsequent alcohol use was not clearly defined. Crum [68] reported that dropping out of high school was associated with a two-fold greater risk of an alcohol use disorder (RR adjusted for sex: 2.1, 95% CI: 1.0 to 4.4, p < 0.05). Even after removing from the sample participants with a report of early onset problem drinking, the results were similar, except for students with a GED whose risk had decreased. However, results should be considered as of high risk of bias due to differential attrition. The association between educational attainment and subsequent alcohol use seemed to be more precarious for students who continued their academic career after high school as they were more likely to become current drinkers than their peers who finished their educational career after high school graduation or who dropped out [44,69]. In the same way, Lee [64] did not observe a significant relationship between high school dropout and subsequent alcohol use. Earlier studies detected higher rates of alcohol use and binge drinking among male high school dropouts only [63].

### Internalizing disorders

Compared to high school graduates, dropouts reported significantly more depression at the time of the expected graduation [70]. However, this association was significant for dropouts with low parental support only. Benjet [27,28] also reported that high school dropouts were significantly more likely to have a mood disorder (OR adjusted for socio-demographic characteristics and service use: 2.16; 95% CI: 1.49 to 3.13; p < 0.05) and Daniel [58] reported that they were at considerable risk for suicidal ideations (OR: 11.03; p < 0.001). In a recent study, Ohayon identified comparable prevalence rates for depressive symptoms among Irish students, youth who work and so-called NEETs (“not in education, employment or training”) [71]. However, they noted an increased probability of developing depressive symptoms when participants worked and had dropped out of school (OR adjusted for age, gender and weight: 2.9; 95% CI: 1.3 to 6.5, p < 0.01).

Surprisingly, there was no significant difference between dropouts and graduates regarding anxiety at the moment of expected graduation. However, two years later, dropouts reported significantly more anxiety than graduates [70]. All those results should be considered with caution as each of the three studies was rated unclear regarding risk of bias.

### Externalizing disorders

After controlling for socio-demographic characteristics, Williams [72] reported that for both, higher and lower achievers, dropping out of high school was significantly related to experiencing externalizing disorders (OR adjusted: 1.69; 95% CI: 1.02 to 2.80; p = 0.043 and OR adjusted: 1.93; 95% CI: 1.14 to 3.30; p = 0.015 respectively). This finding was confirmed by Benjet [27,28] who observed that high school dropouts, compared to in-school youth, had significantly higher odds for any impulse control disorder (OR adjusted for socio-demographic characteristics and service use: 1.44; 95% CI: 1.09 to 1.91; p < 0.05).

### Addressing gender differences

Only 10 of the 51 studies investigated the association of mental disorders and high school dropout by gender.

Regarding adolescent substance use as a predictor of school dropout, results addressing gender differences presented some discrepancy. According to Horwood [36], there were no effect modifications by sex, whereas Krohn [63] and Legleye [26] did detect differential effects of substance use on school dropout for males and females. After controlling for potentially confounding factors, early substance use was significantly related to school dropout for males only [63]. In contrast, Legleye found that girls who reported early consumption of cannabis seemed to be more likely to drop out of high school than boys, considering the adjusted odds ratio of 3.41 (95% CI: 1.89 to 6.13) for girls versus 2.05 (95% CI: 1.41 to 2.99) for boys.

When considering the other direction of the association, high school dropouts reported higher rates of tobacco and cannabis use for both boys and girls, whereas subsequent binge drinking had been observed for male dropouts only [63].

The same had been observed for internalizing disorders. Miech [10] suggested that the effects of mental disorders on educational attainment were similar for men and women whereas other authors observed effect modifications by sex [5,57]. Indeed, in a large longitudinal study, Fletcher detected a significant association between major depression and high school dropout for girls only. The same had been observed for anxiety disorders that seemed to be the most impeding for educational attainment among girls. In contrast, male school dropouts reported significantly more conduct disorders [5].

### Mediating factors of the association

In his review, Rumberger stressed the need to investigate more comprehensive causal models of early school leaving that would identify relevant influences, their interrelationship as well as their cumulative impact on educational attainment [2]. In a later review, Rosenthal deplored that research on non-school correlates of dropout lacked a coherent theoretical framing and thus findings often displayed a variety of specific risk factors without thoroughly considering potentially mediating factors [73]. Confounding factors explored by included records could be divided into three major categories including socio-demographic, family and school-related factors. As can be seen in Figure 2, 49% of included references could be estimated to provide appropriate control for potentially confounders by considering factors from at least two of the three cited domains. Nine studies even considered all three domains [15,36,51,52,54,57,59,63,69] with four of them also being rated as of low risk of bias. These four studies of estimated high quality all investigated the relationship between substance use and educational attainment. 16 other studies only considered one of the cited domains, most frequently socio-demographic characteristics (13 studies) whereas another ten studies only controlled for gender [65,68] or did not provide any multivariable analyses with statistical controls at all [30,31,34,53,60-62]. Only one of the latter studies was methodologically rated as of low risk of bias [34], whereas the risk of bias was estimated as unclear for four studies [30,31,60,62] and as high for the remaining two [65,68]. Thus, results may overestimate the impact of mental health problems on subsequent school dropout as other potentially confounding factors had been omitted.

As detected in early dropout research, socio-demographic characteristics still interfered with educational attainment. In particular, socio-economic status was confirmed as a mediating factor of the relationship between mental disorders and subsequent school dropout [11,29,56,57].

In a cross-sectional study, LeCook reported that the association between psychiatric disorders and subsequent educational attainment varied with race or ethnicity. However, they did not consider the socio-economic situation as a potentially confounding factor, which may induce an omitted bias [41].

Another factor observed to impact the magnitude of the mental health – dropout association was the age of onset for psychiatric disorders. As seen above, several studies confirmed that the negative impact of psychiatric disorders on high school completion was even stronger when the disorder occurred early in life [26,36,49,62].

Valuable and differentiating results regarding co-occurring disorders were presented in the work of McLeod [15] who considered the effect of different combinations of mental disorders on educational attainment. They found out that depression in and of itself was much less consequential for academic achievement than were attention problems, delinquency and substance use, problems known to interfere with school activities and teacher authority. Even in the absence of additional problems, youth who experienced any one of these externalizing problems had lower academic attainment, with substance use having the most consistent negative effect. These observations rose the question as to whether behavioural traits predict educational attainment beyond a reduced academic aptitude. McLeod confirmed this hypothesis by detecting a significant negative impact of substance use and delinquency on educational attainment after controls had been made for grade point average (GPA) as a proxy for academic achievement, thus confirming the results of McCaffrey [54]. In turn, Fletcher who had also used data from the National Longitudinal Survey of Adolescent Health, showed that the odds of depression were reduced by nearly 40% for each point increase in GPA. Considering academic achievement as an endogenous variable, Fletcher assumed that the total effect of depression on educational attainment may be reduced as part of the effect operated through decreases in the GPA [57]. The reciprocal effect of academic achievement and depressive symptoms had also been noted by Quiroga [32] who observed that youth combing a history of grade repetition and depression were most at risk to drop out of high school [32]. As studies including academic confounders had very different foci varying from grade repetitions [5,26,32], school characteristics [50,51], attitudes [63] and grades or test scores as a proxy for academic performance [10,15,52,54,57], a direct comparison of results was difficult.

Family variables including family composition, family functioning, attachment and parental adjustment were considered by 52% of the references with an important focus on family composition (20 out of 26 studies). Only nine studies evaluated potentially confounding factors related to family functioning and attachment. Family and peer influence regarding substance consumption were found to mediate the association between cannabis use and high school dropout [54]. Strong parental support also seemed to be protective against the onset of depression after dropping out of high school [70].

## Discussion

Included references demonstrated a consistent focus in dropout research. Indeed, over half of the records investigated the relationship between substance use and early school leaving. This association had already been depicted in earlier reviews [17,36,74] but its relevance is still highly topical as early substance use can be considered having a unique impeding effect on educational attainment. Indeed, after controlling for socio-demographic, family and academic factors, adolescents who began to use cannabis before the age of 16 were up to five times more likely to drop out of secondary school than their peers who did not consume any drugs. Surprisingly, compared with total abstinence, cannabis experimentation only seemed to enhance high school graduation. As cannabis experimentation is nowadays very common among adolescents, this behaviour may reflect successful peer integration and thus a positive social experience that may in turn be protective for educational attainment [26]. However, patterns of use should be followed with caution as the transition from experimentation to frequent use equals a tightrope walk and we noted a clear dose–response relationship, where the odds of dropping out increased with the frequency of use. Indeed, even after excluding references with an estimated unclear or high risk of bias as well as studies without sufficient confounding (at least two of three major domains), the deleterious unique effect of early cannabis use remained a strong predictor of early school leaving [5,10,36,40,52,63,67,75]. The same had been observed for smoking [48].

Earlier studies reported a significant association between school dropout and subsequent cannabis use whereas more recent work did not confirm a significant association [42,52]. Such discrepant findings may be explained by the progressive detection and consideration of potentially confounding factors that in turn reduced the effect of school dropout on subsequent substance use to insignificance. However, early school leavers who already consumed cannabis were more at risk of developing a substance use disorder.

Besides illicit drugs, smoking was strongly related to secondary school dropout whereas alcohol was not. Furthermore, regarding alcohol, it was the students who continued their academic career, who were more at risk of becoming current drinkers than their peers who had dropped out. These observations regarding cannabis use, smoking and alcohol consumption remained valid when references estimated as of low risk of bias and with complete confounding were considered exclusively.

Another strong and independent predictor of secondary school dropout were so-called externalizing disorders referring to disruptive behaviour, attention problems and inadequate social adjustment. Several authors confirmed a significant association with odds ratios varying from 1.89 [39] up to 6.74 [58]. The observed wide range of odds ratios reflected the heterogeneous control for potentially confounding factors. After excluding studies with an unclear or high risk of bias as well as those referring to incomplete confounding (only one category or no confounding at all), the magnitude of the association decreased but remained significant with odds ratios varying from 1.89 [39] up to 3.35 [10]. Attention deficit disorder with or without hyperactivity was also reported to be particularly impeding for educational attainment as it is a condition that implies cognitive and behavioural symptoms. This observation was confirmed by two studies estimated as having a low risk of bias, with odds ratios decreasing from 2.7 [34] to 2.06 [39] as a consequence of increased confounding. Furthermore, when considering academic ability as a mediating variable, the association between attention deficit disorder and school dropout became insignificant [10]. However, the burden of symptoms seemed to decrease with age as they were no longer associated with later occupational outcomes.

In the same way, mood disorders and, to a lesser extent, anxiety disorders were significantly related to subsequent school dropout, in particular among girls. However, when referring exclusively to studies with an estimated low risk of bias, the association between internalizing disorders and educational attainment seemed to be strongly mediated by co-occurring disruptive behaviour problems and by academic achievement, thus becoming insignificant [10,39].

The observed gender differences may reflect a different expression of symptoms or coping style among boys and girls. Educational attainment among girls may be more affected by symptoms that involve a loss of motivation, cognitive slowness or inhibition, whereas achievement among boys may by more affected by non-cognitive traits such as aggressive behaviour or restlessness. Therefore we consider it crucial to further address gender differences in dropout research related to the interplay of mental health problems and academic achievement in order to adapt intervention strategies.

Internalizing disorders were also found to be an outcome of secondary school dropout, above all mood disorders and suicidal ideations. Anxiety disorders did not develop soon after the dropout but only after a few years. Indeed, after a frustrating and fearful school career, dropping out can first be considered as a relief. However, as vocational opportunities are very limited and unstable for young people with low qualifications, expected living conditions risk being precarious, thus leading to the onset of anxiety and mood disorders.

### Limitations

The present review provides an extensive insight into dropout mental health research over the last 20 years thus updating on previous collective work [2,17,73,74]. However, some limitations have to be considered. First, the selection of references only targeted scientific work that had been published in peer-reviewed journals and indexed in the relevant databases. Thus, important references that had not been published or issued in grey literature may have been missed.

Considering the methodological differences among the various empirical studies addressing secondary school dropout as well as the diversity of potentially confounding factors, it seemed unfeasible to combine their information into a unique understanding of the problem by providing pooled estimates of the mental health – dropout association as well as a sensitivity analysis to compare results issued from studies with different risk of bias assessment [3]. However, the present systematic review aims to induce a certain degree of methodological homogeneity that allows a comparison of findings in order to shape a global but comprehensive picture of the dropout-mental health association. Therefore, we focused on psychiatric disorders as defined by DSM criteria, with exceptions made for patterns of substance use. This approach may be considered restrictive or a source of potentially missed information, but if we consider the 19 references that were excluded because they used diagnostic instruments referring to broader symptom criteria, their findings generally matched the results presented above, with few exceptions to be mentioned. Depressive symptoms only, in contrast to major depression, were not significantly related to secondary school dropout suggesting that a certain duration and functional impairment had to be reached before symptoms began to impede educational outcomes [76]. In a case control study, Trampush [77] found that cognitive ability, substance use and contact with the biological father were significantly related to school dropout, but that the magnitude of this association did not differ among adolescents with and without ADHD. If possible, with the objective to induce some homogeneity in presenting the results of included references, we indicated the magnitude of the mental health – dropout association by odds ratios, together with their confidence intervals. However, for some studies, the confidence interval could not be retrieved based on available data [53,58]. Furthermore, a standardized risk of bias assessment had been conducted in order to add validity to the presented structured summary of evidence regarding the bidirectional association between mental health and early school leaving. In addition, a qualitative comparative analysis was conducted to explore whether major conclusions per disorder category varied with the overall risk of bias evaluation and the extent of considered confounding. To enhance overall transparency and clarity of the present work, we adhered to the PRISMA guidelines for reporting systematic reviews [25]. The completed PRISMA checklist was provided as an Additional file 6.

## Conclusions

According to the present systematic literature review, disruptive behaviour and substance use disorders seemed to be the most impeding for educational attainment whereas internalizing disorders had a weaker effect on school dropout. Future research targeting the sequence of the downward spiral of psychological symptoms and academic experiences could build on previous work evaluating the effects of early behaviour disturbances. Duncan identified childhood conceptual and procedural competencies as an independent predictor of later academic achievement, to a stronger extent than communication and social skills [78]. In continuity, Breslau confirmed that only early attention problems, when controlling for co-occurring internalizing and externalizing problems, seemed to have a negative impact on later academic achievement [75]. An explanatory hypothesis to be explored in a longitudinal design could be that children with reduced conceptual and procedural competencies would experience more difficulties and frustrations regarding educational success and thus engage in externalizing problem behaviour, whereas children with reduced social skills may develop an internalizing coping style that seems to be less impeding for educational attainment.

On the other hand, internalizing disorders were reported to be a consequence of early school leaving, considering the precarious educational and professional opportunities of young people having less than secondary education.

Only few studies explored the question of gender differences and among those that had, the findings were discrepant, even for studies with an estimated low risk of bias and adequately addressed confounding.

Over time, there was a progressive awareness to consider potentially confounding factors of the relationship between mental health issues and school dropout. However, the present review depicted relatively few factors as having some mediating influence. They included socio-economic characteristics, the age of onset for mental disorders, academic performance and family support.

A striking observation concerned the nature of confounding factors that were included in the models. We detected a clear focus on individual, family- and school-related variables that could be considered immutable because inherent to the student or his environment. Characteristics including cognitive ability, family composition, socio-economic situation or school location provide a valuable epidemiological input to early detection strategies of students at risk of dropout, but they cannot be targeted by intervention programs aimed to reduce early school leaving. Future research should consider these observations and focus on alterable mediating factors such as school climate, family functioning or individual coping styles in order to support the development and implementation of effective policies covering all three levels of action: prevention, intervention and compensation.

## Additional files

Additional file 1:
**Included references (mental disorders as predictor of school dropout).**
Additional file 2:
**Included references (school dropout as predictor of mental disorders).**
Additional file 3:
**References without DSM criteria - Addendum.**
Additional file 4:
**Checklist summarising compliance with MOOSE guidelines, applied to Horwood et al., 2010.**
Additional file 5:
**Risk of bias for all included studies (n=51).**
Additional file 6:
**PRISMA 2009 Checklist.**
