# Supplementary material for: The downward spiral of mental disorders and educational attainment: a systematic review on early school leaving
Source: BMC Psychiatry. 2014 Aug 27;14:237. doi: 10.1186/s12888-014-0237-4 (PMC4244046; doi:10.1186/s12888-014-0237-4)
Supplement: Additional file 1: — Included references (mental disorders as predictor of school dropout). [file 12888_2014_237_MOESM1_ESM.pdf]

Additional file 1 Included references (mental disorders as predictor of school dropout)

Reference	Study design	Sample<br>**N, age range, gender**	Dropout definition<br>**HS: High school**<br>**GED: General Educational Development**	Hypotheses	Disorder types	Evaluation of disorders	Confounding factors	Significant results<br>**ORadj: adjusted odds ratio**<br>**CI: confidence interval**<br>**RR: relative risk**	Overall risk of bias<br>**(for details, see additional file 4)**
Hemphälä & Hodgins<br>2014<br>Sweden	longitudinal, 1 wave	N=147<br><br>mean age at wave 1: 16.8 years<br><br>61 male & 86 female	Having less than 7 years of education	Conduct disorder symptoms prior to age 15 -> school dropout	Conduct disorder	Psychopathy Checklist - Youth Version (PCL-YV)<br><br>Schedule for Affective Disorders and Schizophrenia for school-aged children (K-SADS)<br><br>Structured Clinical Interview for DSM IV disorders (SCID)	not applied	Conduct disorder symptoms were significantly associated with school dropout (ORadj=1.35 (95%CI:1.08-1.69), p-value=0.009.	unclear
Quiroga et al.<br>2013<br>Montreal, Canada	longitudinal, 3 waves	N=493<br><br>mean age: 12.5 years<br><br>265 male & 228 female	Noncompletion of secondary education	Depressive disorder -> academic competence/achievement -> school dropout	Depressive disorder	Inventory to Diagnose Depression (IDD)	Gender<br>Parental education<br>Grade retention<br>Academic competence<br>Academic achievement	The results showed an indirect relationship between depression and dropout through self-perceived academic competence (standardized indirect coefficient=0.05), indicating that the probability of dropping out of school was 5% higher each time that depression symptoms increased by one standard deviation.	unclear
Quiroga et al.<br>2012<br>Montreal, Canada	longitudinal, 3 waves	N=453<br><br>mean age: 12.5 years<br><br>52% male & 48% female	Noncompletion of secondary education	Grade retention -> depressive disorder -> school dropout	Depressive disorder	Inventory to Diagnose Depression (IDD)	Gender<br>Parental education<br>Grade retention<br>Academic competence<br>Academic achievement<br>Anxiety<br>School rebelliousness<br>Friends school engagement<br>Student teacher conflict	Students who had repeated a grade in primary school were 5.54 times more likely to drop out (95%CI: 2.46-12.46).<br>Depression symptoms in the seventh grade were also associated with a higher risk to drop out (ORadj=2.75, 95%CI: 1.18-6.42), p-value<0.01).<br>The likelihood of dropping out for students with a history of grade retention who also reported depressive symptoms in 7th grade was 7.26 times higher than for those who did not report depression (95%CI: 1.46-36.17, p-value<0.01). No gender differences noted for the reported interplay.	high
McLeod [15]<br>2012<br>USA	longitudinal, 4 waves<br><br>National Longitudinal Study of Adolescent Health (NLSAH)	N=6315<br><br>9-12th grade<br><br>46% male & 54% female	Educational milestones:<br>*HS dropout*<br>*GED*<br>*HS graduation*<br>*Technical training*<br>*College entry*<br>*College graduation*	Mental disorders -> Educational attainment	Depressive disorder<br>Attention deficit disorder with/ without hyperactivity<br>Delinquency<br>Substance use	Center for Epidemiological Studies Depression Scale (CES-DC)<br>SNAP-IV	Age<br>Gender<br>Race<br>Socioeconomic status<br>Family structure<br>Grade Point Average (GPA)<br>Combination of mental health problems<br>Academic aptitude	All 4 disorder categories were associated with receiving a lower degree when considered alone.<br>When considering the results of the full model (including GPA) the coefficients for delinquency and substance use were reduced by about half, but remained significant, whereas the coefficient for attention problems became marginally insignifiant (p-value 0.093).<br>Adolescents who experienced more than one mental health problem generally achieved a lower degree than youth who experienced only one problem.<br>Co-occurring attention problems heightened the risk of low educational attainment	unclear
Borges et al.<br>2011<br>Mexico City, Mexico	cross-sectional	N=2362<br><br>18-65 years	Noncompletion of secondary education	Mental disorder -> school dropout	Mood disorders<br>Anxiety disorders<br>Impulse control disorders<br>Substance use disorder	Composite International Diagnostic Interview (CIDI)	Childhood traumatic events<br>Childhood neglect<br>Parental mental illness<br>Family disruption<br>Low parental education<br>Age<br>Gender<br>Sociodemographic variables	Compared with having no disorder, generalized anxiety and post-traumatic stress disorder significantly reduced the odds for dropping out of highschool. In contrast, drug abuse and dependence increased the odds for dropout.<br>The proportion of people dropping out of secondary school would only decrease by 0.53% in the absence of mental disorders, suggesting a strong impact of socioeconomic or cultural adversities.	low
Porche et al. [30]<br>2011<br>USA	cross-sectional retrospective<br><br>Collaborative Psychiatric Epidemiological Surveys (CPES)	N=2532<br><br>21-29 years	Noncompletion of HS	Early childhood trauma -> Childhood psychiatric disorders -> HS dropout<br><br>Menatl health services use -> School attendance	Major childhood trauma<br>Depressive disorder<br>Anxiety disorder<br>Post traumatic stress disorder<br>Substance use disorder<br>Conduct disorder	Composite International Diagnostic Interview (CIDI)	Gender<br>Race/ethnicity<br>Parental education level<br>Nativity<br>Early youth employment<br>Early pregnancy	Substance use disorder (ORadj=2.48 CI=1.30-4.74) and conduct disorder (ORadj=2.38 CI=1.43-3.96) were significantly associated with HS dropout.<br>After adjusting for psychiatric disorders, the OR of dropping out associated with childhood trauma was no longer significant (ORadj=1.36 CI=0.98-1.89).<br>Services use was not considered a mediator of the relation between childhood trauma and dropout (ORadj=0.95 CI=0.57-1.59).	unclear

Reference	Study design	Sample<br>**N, age range, gender**	Dropout definition<br>**HS: High school<br>GED: General Educational Development**	Hypotheses	Disorder types	Evaluation of disorders	Confounding factors	Significant results<br>**ORadj: adjusted odds ratio<br>CI: confidence interval<br>RR: relative risk**	Overall risk of bias<br>**(for details, see additional file 4)**
Kent et al. [27]<br>2011<br>Pittsburgh, USA	case control study<br>case: ADHD diagnosis<br>control: no diagnosis<br>matching: age, gender, race, parent education level<br><br>longitudinal, 4 waves<br>Pittsburgh ADHD Longitudinal Study (PALS)	N=539 (326 cases/213 controls)<br><br>mean age ADHD: 17.6 years<br>mean age control: 17.1 years<br><br>**100% male**	Noncompletion of HS	Attention Deficit Disorder with Hyperactivity (ADHD) -> HS dropout	Attention Deficit Disorder with Hyperactivity (ADHD)<br>Oppositional Defiant Disorder (ODD)<br>Conduct disorder (CD)	Childhood clinical evaluation<br>Disruptive Behavior Disorder rating Scale (DBD)	Parental education level<br>IQ	Adolescents with ADHD were significantly more likely to drop out of HS than adolescents without ADHD (ORadj=8.14 p<0.01).<br>However, symptom severity of ADHD, ODD and CD for adolescents with ADHD were not significant predictors of dropout.	high
Breslau et al. [31]<br>2010<br>USA	longitudinal, 2 waves<br><br><br>National Epidemiological Survey of Alcohol and Related Conditions (NESARC)	N=29662<br><br>>= 18 years<br><br>48% male & 52% female	Noncompletion of HS on time<br>Only attaining GED or Late HS graduation (>= 19 years)	Early onset psychiatric disorder -> Failure to complete HS on time	Mood disorder<br>Anxiety disorder<br>Conduct disorder<br>Attention Deficit Disorder with Hyperactivity (ADHD)<br>Substance use disorder (SUD)	Alcohol Use Disorders and Associated Disabilities Interview Schedule (AUDADIS)	Childhood adversities<br>Age<br>Gender<br>Nativity<br>Region of the US<br>Race/ethnicity	Adjusting for childhood adversities, socio-demographic variables and co-occuring disorders, only conduct disorder (ORadj=1.89 CI=1.57-2.26) and all 3 types of ADHD were significantly associated with noncompletion of HS on time. Among ADHD types, the association was strongest for the combined type (ORadj=2.06 CI=1.66-2.56).<br><br>Among substance use disorders, nicotine dependence (ORadj=1.52 CI=1.13-2.03) and drug use or dependence (ORadj=1.34 CI=1.11-1.62) were significantly associated with HS failure. No association between alcohol and HS failure.	low
LeCook et al. [33]<br>2010<br>USA	cross-sectional<br>retrospective<br><br>Collaborative Psychiatric Epidemiological Surveys (CPES)	N=5839<br><br>>= 18 years	Noncompletion of HS	Early onset psychiatric disorder -> HS dropout with a vriation of the association by race/ ethnicity	not reported	Composite International Diagnostic Interview (CIDI)	Age<br>Gender<br>Marital status	Individuals with early onset psychiatric disorders were more likely to have less than a HS education. After adjustment, Latinos (40%) and Blacks (31%) were more likely than Whites (15%) to drop out.	unclear
McCaffrey et al. [43]<br>2010<br>South Dakota, USA	longitudinal, 6 waves<br><br><br>ALERT & ALERT Plus<br>(Drug prevention programs)	N=4375<br><br>mean age: 12.36 years<br><br>50% male & 50% female	Noncompletion of HS	Heavy & persistent marijuana use in grades 9 and 10 -> Cognitive impairment -> HS dropout	Heavy & persistent marijuana use	use of marijuana in the past month and 3 or more times in that period	Age<br>Gender<br>Race/ethnicity<br>Family structure<br>Parental education level<br><br>School performance<br>Deviant behavior<br>Attitudes school & family<br>Family influences<br>Peer influences<br>Substance use	Controlling for socio-demographic covariates, the odds of HS dropout are 2.4 times higher for marijuana users compared to nonusers. This association does not change significantly after considering alcohol consumption.<br>But after introducing cigarettes in the model, marijuana use is no longer significantly related to HS dropout, suggesting an omitted variable bias.<br>In contrast to the hypothesis, school performance did not mediate the association between marijuana use and dropout, but family and peer influence did.	unclear
Horwood et al. [28]<br>2010<br>Australia & New Zealand	Meta-analysis of 3 birth cohort studies<br><br>Christchurch Health and Development Study (CHDS)<br><br>Victoria Adolescent Health Cohort Study (VAHCS)<br><br>Mater-University of Queensland Study of Pregnancy and Outcomes (MUSP)	N1=1003-1025 (cohort CHDS)<br>N2=1523 (cohort VAHCS)<br>N3=3768 (cohort MUSP)	Noncompletion of HS	Early onset cannabis use -> HS dropout	Cannabis use	Age of first use:<br><15 years<br>15-17 years<br>never used before age 18	Family socio-demographic background<br>Child cognitive ability<br>Family functioning<br>Child/early adolescent behavioural adjustment	The pooled estimate of the adjusted ORs showed that compared to those who began cannabis use <15 years, those who began at age 15-17 were 1.7 times more likely to complete HS (ORadj=1.7 CI=1.4-2.1, p<0.001), whereas those who had never used cannabis by age 18 had an adjusted OR of 2.9 (CI=1.8-4.6 p<0.001).<br>However, there was evidence of significant heterogeneity of parameter estimates (Cochran's Q(2df)=6.22, p=0.04) reflecting a more modest association for VAHCS.<br>There was no significant gender x age of onset interaction for HS completion.<br>The pooled estimate suggested that the early use of cannabis accounted for 17% of the overall rate of HS noncompletion (PAR).	low

Reference	Study design	Sample<br>N, age range, gender	Dropout definition<br>HS: High school<br>GED: General Educational Development	Hypotheses	Disorder types	Evaluation of disorders	Confounding factors	Significant results<br>ORadj: adjusted odds ratio<br>CI: confidence interval<br>RR: relative risk	Overall risk of bias<br>(for details, see additional file 4)
Goulding et al. [48]<br>2010<br>Atlanta, USA	cross-sectional<br><br>Atlanta Cohort on the Early Course of Schizophrenia (ACES)	N=109<br>18-39 years (mean: 23.1 years)<br>76% male & 24% female<br>**clinical sample**	Noncompletion of HS	Schizophrenia spectrum disorder -> HS dropout <-> Social and clinical variables	Episode of nonaffective psychosis<br><br>Daily nicotine use<br>Weekly alcohol use<br>Daily cannabis use	Structured Clinical Interview for DSM Disorders - Axis I (SCID)	not applied	44% of participants had dropped out of HS which is much higher than in a non-clinical sample (rates of 13-18%).<br>The 2 most common diagnoses for school dropouts were schizophrenia, paranoid type (45.8%) and schizophreniform disorder (18.8%). HS dropouts were significantly younger at time of hospitalisation than HS graduates.<br>HS dropout was significantly associated with daily nicotine use, daily cannabis use, poorer academic performance and poorer overall social functioning.	unclear
Marti et al. [62]<br>2010<br>USA	longitudinal, 8 waves	N=496<br>mean age: 13.5 years<br>**100% female**	Having dropped out prior to age 18	Polysubstance use -> School dropout	Polysubstance abuse symptoms<br>Polysubstance abuse diagnosis<br>Polysubstance: tobacco, alcohol, cannabis, stimulants, downers, inhalants and hallucinogens	Schedule for Affective Disorders and Schizophrenia for school-aged children (K-SADS)<br>*Child Behavior Checklist(CBCL) Externalizing Scale*	not applied	Among early heavy substance users, 31.4% had dropped out of school. Problematic use (OR=5.33 p<0.001), problematic abuse (OR=3.69 p<0.001), late-heavy substance use (OR=4.45 p<0.01) and moderate escalating use (OR=5.56 p<0.01) were significantly associated with school dropout prior to age 20.	high
Legleye et al. [24]<br>2010<br>Metropolitan France	cross-sectional	N=29393<br>age range: 17 years<br>51% male & 49% female	Having dropped out of school at age 17	Cannabis use -> School dropout	Polysubstance use (tobacco, alcohol, cannabis, so-called rare drugs as poppers, hallucinogenic mushrooms, ecstasy, cocaine amphetamines, crack, heroin, LSD)	Age of onset<br><br>Age at the transition to daily use	Socioeconomic status<br>Parents' divorce/separation<br>Grade repetition	Compared with lifetime abstinence, experimentation alone before age 14 was not linked to school dropout whereas experimentation at age 15-16 was associated with school attainment (ORadj=0.80 for boys and 0.64 for girls)<br>School dropout was more common when daily cannabis use was reported early (<14 years) with an ORadj= 2.05 for boys and ORadj=3.41 for girls.<br>The daily consumption of tobacco had a major impact on school dropout for both sexes (ORadj= 2.62 for boys and 2.92 for girls).<br>Experimentation alone with alcohol and cannabis during school years enhanced school attainment compared with total abstinence.	unclear
Lee et al. [44]<br>2009	cross-sectional<br><br>World Mental Health Survey<br><br>7 low- and middle-income (LAMI) countries<br>9 high-income countries	N=41688	Noncompletion of one of the educational milestones:<br>*primary education*<br>*secondary education*<br>*entry to tertiary education*<br>*tertiary education*	Early onset mental disorder -> School dropout	Anxiety disorders<br>Mood disorders<br>Behavior/impulse control disorder<br>Substance use disorder (SUD)	Composite International Diagnostic Interview (CIDI)	Childhood adversities<br>Age<br>Sociodemographic variables<br>Dummy variables for countries' income level	Every disorder category was significantly related to non-completion of secondary school in high-income countries:<br>Any anxiety disorder: ORadj=1.3 CI=1.2-1.4; p<0.01<br>Any mood disorder: ORadj=1.4 CI=1.2-1.6; p<0.01<br>Any impulse disorder: ORadj=2.2 CI=1.8-2.7; p<0.01<br>Any substance use disorder: ORadj=2.8 CI=2.3-3.3; p<0.01<br>Any mental disorder: ORadj=1.4 CI=1.3-1.5; p<0.01<br><br>In low-income countries, only mood disorders (ORadj=1.3 CI=1.0-1.6; p=0.03); impulse disorders (ORadj=1.3 CI=1.1-1,6; p=0.01) and substance use disorders (ORadj=1.5 CI=1.1-2.1; p=0.02 were significantly related to dropout.<br><br>In both, LAMI and high-income countries, the impact of any mental disorder on educational attainment was strongest for the stage of completing secondary education.	unclear
Fletcher [45]<br>2008<br>USA	longitudinal, 3 waves<br><br>National Longitudinal Study of Adolescent Health (NLSAH)	N=13000<br>Grades 7-12<br>wave 1: mean age: 17 years<br>wave 2: mean age: 18 years<br>wave 3: mean age: 22 years<br>47% male and 53% female	Noncompletion of HS<br>Not entering college	Adolescent depression -> HS dropout	Depressive disorder	Center for Epidemiological Studies Depression Scale (CES-D)	Age<br>Gender<br>Grade Point Average (GPA)<br>Maternal education<br>Family income<br>Marital status<br>Ethnicity/race	No significant association between depression and school dropout in males, but in females: depressed girls were 3.5 percentage points less likely to graduate from HS and 6 percentage points less likely to enroll in college than non-depressed girls. The latter association was even stronger for girls from relatively rich families. Intact family structure, family income and maternal education were positively related with college enrollment.<br><br>Wave 1 GPA was negatively related to depression, reducing the odds of depression by nearly 40% for each point increase in GPA.	unclear

Reference	Study design	Sample<br>**N, age range, gender**	Dropout definition<br>**HS: High school<br>GED: General Educational Development**	Hypotheses	Disorder types	Evaluation of disorders	Confounding factors	Significant results<br>**ORadj: adjusted odds ratio<br>CI: confidence interval<br>RR: relative risk**	Overall risk of bias<br>**(for details, see additional file 4)**
Breslau et al. [32]<br>2008<br>USA	longitudinal, 2 waves<br><br>National Comorbidity Survey Replication (NCS-R)	N=5692<br><br>>= 18 years	Noncompletion of one of the educational milestones:<br>*primary education*<br>*highschool*<br>*college entry*<br>*college graduation*	Early onset psychiatric disorder -> Failure to complete HS on time	Anxiety disorders<br>Mood disorders<br>Impulse control disorder<br>Substance use disorder (SUD)	Composite International Diagnostic Interview (CIDI)	Childhood adversities<br>Age<br>Gender<br>Race/ethnicity	Compared to having no disorder, having exactly 1 disorder is not significantly associated with higher odds of school dropout, but having two (OR=1.4) or 3 (OR=3.5) disorders is significantly associated with higher odds of dropout.<br>Of the 17 disorders considered, 12 were significantly related to HS dropout.<br>Any anxiety disorder: ORadj=1.3 CI=1.0-1.5; p=0.02<br>Any mood disorder: ORadj=1.5 CI=1.1-1.9; p=0.003<br>Any impulse disorder: ORadj=1.8 CI=1.4-2.4; p<0.001<br>Any substance use disorder: ORadj=2.9 CI=2.1-4.0; p<0.001<br><br>The proportion of people dropping out of HS would decrease by 10.2% in the absence of mental disorders.	low
Degenhardt et al.<br>2007<br>Victoria, Australia	longitudinal, 8 waves<br><br>Victoria Adolescent Health Cohort	N=1520<br><br>wave 1: mean age: 14.9 years<br>wave 8: mean age: 24.1 years		Adolescent amphetamine use -> Noncompletion of secondary school (year 12)	Anxiety and depression symptoms<br>Amphetamine use<br>Cannabis and alcohol use<br>Tobacco use<br>Antisocial behaviour	Self-reported measures for the use of amphetamine, alcohol, cannabis & tobacco	Gender<br>Parental smoking<br>Parental separation	Participants reporting symtoms of anxiety or depression were at over twice the risk of concurrent amphetamine use (ORadj=2.4 CI=1.7-3.4; p<0.001)<br>Frequent cannabis use increased the likelihood for amphetamine use: OR=4.6 CI=2.2-10.0; p<0.001 for less than weekly use and ORadj=27 CI=11.0-68.0; p<0.001 for daily use.<br>Early-onset amphetamine users had higher rates of school dropout: ORadj=2.7 CI=1.8-3.9; p<0.001<br>When adjusting for cannabis use, the assocaition was decreased: ORadjcann=1.6 CI=1.0-2.7; p=0.05<br>There was no evidence of effect modification by sexe.	high
Bohon et al. [41]<br>2007	longitudinal, 7 waves	N=240<br><br>46% male & 54% female	Noncompletion of HS	Maternal depression -> HS Dropout	Maternal Depression<br>Externalizing symptoms<br>Adolescent substance use<br>Adolescent depression	Structured Clinical Interview for DSM Disorders - Axis I (SCID)<br>Schedule for Affective Disorders and Schizophrenia for school-aged children (K-SADS)<br>*Child Behavior Checklist(CBCL)*	Children's IQ<br>Mother's educational status<br>Socioeconomic status<br>Adol. externalizing symptoms<br>Adolescent substance use<br>Adolescent depression	Substance use before grade 9 (OR=2.97 p<0.001) and extrenalizing behavior in grades 6-8 (OR= 1.05 p<0.05) were significantly related to HS Dropout<br>A higher IQ was associated with lower odds of dropping out for offspring of never or moderately depressed mothers but not among offspring of mothers with chronic or severe depression	low
Barbaresi et al. [26]<br>2007<br>Rochester,Minnesota,USA	case control, longitudinal<br><br>case: children with ADHD<br>control: children without ADHD<br>matching: age, gender	N=1110<br>370 cases & 740 controls<br><br>median age at last follow-up: 18.4 years for case and 18.3 for controls<br><br>cases: 75% boys & 25% girls	Not graduating from HS, with<br>or without pursuing a GED<br>or an adult diploma	ADHD -> HS dropout	Attention Deficit Disorder with Hyperactivity (ADHD)	Incident ADHD diagnosis was based on several steps<br>1. Review of school records<br>2. Review of medical records<br>3. ADHD questionnaire<br>4. Clinical diagnosis	Matching criteria (age, gender)	Among the remaining 301 AD/HD cases, 22.9% dropped out of HS compared to 10.0% of the remaining 609 controls.<br>Participants with AD/HD were 2.7 times (CI:1.8-3.9; p<0.001) more likely to drop out than controls, based on an analysis adjusting for gender and year of birth.	low
Daniel et al. [46]<br>2006<br>USA	longitudinal, 4 waves	N=188<br><br>15.0-15.9 years; mean: 15.4<br><br>56% male & 44% female	Noncompletion of HS<br>Pursuing a GED<br>Receiving vocational training<br>Incarceration	Suicidality -> School dropout	Major depressive disorder<br>Conduct disorder/Oppositional defiant disorder<br>Substance use disorder (SUD)<br>Suicidal behaviors	Schedule for Affective Disorders and Schizophrenia for school-aged children (K-SADS)	Age<br>Gender<br>Socioeconomic status<br>Race/ethnicity	Major depression (ORadj=3.38; p=0.04); conduct disorder (ORadj=6.74; p=0.001) and reading status (ORadj=5.81; p=0.002) were significantly related to school dropout.<br><br>Suicidality was strongly associated with school dropout (ORadj=7.29; p<0.001). Similarly, dropout was related to an increased incidence of suicidality (ORadj=11.03; p<0.001)	unclear
Kogan et al. [52]<br>2005<br>USA	longitudinal, 2 waves<br><br>Data from the National Educational Longitudinal Study (NELS)	N=1762<br><br>grade 8-12<br><br>49% male & 51% female<br><br>**African American youth**	Noncompletion of HS	Substance use -> HS dropout	Alcohol use disorder<br>Marijuanan use<br>Cigarette use	Frequency of use during the past 30 days for alcohol and cannabis<br>Daily use for cigarettes	Gender<br>Parenthood<br>Socioeconomic status<br>Time since dropout<br>Behavior problems in school<br>Academic problems<br>Conventional peers<br>Positive life orientation	18% of the participants dropped out of school.<br>Youth who used marijuana at T1 were 1,6 times more likely to drop out than were non-users. For each unit level of increase in problem behaviour, youth were 2.1 times more likely to drop out.<br>Dropout partially mediated the association between T1 problem behaviour and T2 substance use.	low

Reference	Study design	Sample<br>**N, age range, gender**	Dropout definition<br>**HS: High school**<br>**GED: General Educational Development**	Hypotheses	Disorder types	Evaluation of disorders	Confounding factors	Significant results<br>**ORadj: adjusted odds ratio**<br>**CI: confidence interval**<br>**RR: relative risk**	Overall risk of bias<br>**(for details, see additional file 4)**
Mcshane et al. [57]<br>2004<br>Sydney, Australia	longitudinal, 3 waves	N=117<br><br>12-21 years, mean age at follow-up: 16.2 years<br><br>**clinic-referred sample for school refusal**		Mental disorder -><br>Not attending a regular state or fee-paying school 3 years later	Anxiety disorder<br>Mood disorder	*Child Behavior Checklist (CBCL)*<br>Global Assessment of Functioning (GAF)<br><br>Center for Epidemiological Studies Depression Scale for Children (CES-DC)	Gender<br>Hospitalization status<br>Age<br>Onset and duration of school refusal<br>Number of school attended<br>Use of medication<br>Family composition<br>Family psychiatric history	After treatment for school refusal, 70% had improved occupational outcomes, with no differences between treatment status.<br>When all significant variables were entered in a logistic regression,major depression (ORadj=0.28; CI=0.11-0.70) and dysthimia (ORadj=0.26; CI=1.10-0.26; CI=0.10-0.67) were predictive of poor occupational outcomes at 6 months follow-up, but not at 3 years.<br>Social phobia (ORadj=0.17; CI=0.04-0.70) was the only predictor of poor outcomes at 3 years follow-up.	high
Fergusson et al. [42]<br>2003<br>Christchurch, New Zealand	Birth cohort, 21 waves<br><br>Christchurch Health and Development Study (CHDS)	N=1265<br><br>15-25 years<br><br>50% male & 50% female	HS Dropout: having left school by age 18 without achieving at least one pass grade in School Certificate examinations	Cannabis use -> HS dropout	Frequency of cannabis use		Socio-demographic factors<br>Changes of parents<br>Childhood adversities<br>Parental attachment<br>Parental adjustment<br>Childhood cognitive ability<br>Gender<br>Cigarette smoking<br>Novelty seeking<br>Deviant peer affiliations<br>Childhood behavior problems<br>Conduct problems	*Accumulative cannabis use -> school dropout:*<br>After adjustment for sign. confounding factors, people who had used cannabis on more than 100 times by age 16 had odds of HS dropout that were 3.7 (1.8-7.5; p<0.001) times higher those who had not used cannabis.	low
Ensminger et al.<br>2003<br>Chicago, USA	longitudinal, 4 waves<br><br><br>Woodlawn Study	N=952<br><br>6-42 years<br><br>**Urban African Americans**	Noncompletion of HS<br>**(Having a GED is NOT classified as dropout)**	Mother's psychological distress -> Major depressive disorder of adult children<br><br>Mother's psychological distress -> Educational attainment of adult children	Major depressive disorder (MDD)	Composite International Diagnostic Interview (CIDI)	Poverty<br>Mother's education<br>Teen motherhood<br>High residential mobility<br>Family composition<br>Mother's physical health<br>Children's social adaptation<br>Children's psychological symptoms	Persistant maternal distress is significantly related to educational attainment for sons, but not for daughters (ORadj=2.29 CI=1.20-4.37; p<0.05) VS ORadj=1.12 CI=0.51-2.49).<br>Persistant maternal distress is also significantly related to MDD, but this time only for daughters (ORadj=2.63 CI=1.15-6.03; p<0.05).	high
Van Ameringen et al. [47]<br>2003	cross-sectional retrospective	N=201	Noncompletion of HS	Anxiety disorder -> School dropout	Panic disorder/agoraphobia<br>Generalized social phobia	Structured Clinical Interview for DSM Disorders - Axis I (SCID)<br>Beck Depression Inventory(BDI)<br>Fear Questionnaire<br>Sheehan Disability Scale (SDS)	not applied	Of the 201 patients, 52 (25.9%) did not complete HS and 98 (48.8%) left school prematurely (HS/college/university).<br>diagnosis of GSP versus 46 of the 103 patients who did<br>There was a mrginally significant association between	unclear
Lynskey et al. [63]<br>2003<br>Victoria, Australia	longitudinal, 7 waves	N=1535<br><br>15-21 years	Noncompletion of secondary schooling (year 12)	Regular cannabis use -> School dropout	Weekly cannabis use	Clinical Interview Schedule-Revised (CIS-R)	Place of birth<br>Location of school<br>Parental separation<br>Parental education<br>Cigarette smoking<br>Alcohol consumption<br>Antisocial behavior<br>Psychiatric morbidity	After controlling for potentially confounding factors, young people who reported weekly cannabis use were at increased risk for dropping out of school. In year 10, they were 5.6 (CI=2.0-15.0) times more likely to leave school than nonabusers and 2.2 times in year 11 whereas in year 12, the association was not significant (ORadj=1.1 CI=0.4-2.9).	high
Vander Stoep et al. [11]<br>2003<br>New York, USA	longitudinal<br><br>Children in Community Study (CICS)	N=967<br><br>52% male & 48% female	Noncompletion of secondary school	Psychiatric disorder -> School dropout	Anxiety disorder<br>Depressive disorder<br>Substance abuse<br>Disruptive disorders		Socioeconomic status	43.8% of the failure to complete school could be attributed to psychiatric disorders. When controlling for socio-economic status, the proportion of school dropout attributable to psychiatric disorders was 44% in the lower SES stratum and 61% in the upper stratum.	unclear
Ellickson et al. [40]<br>2001<br>California, Oregon, USA	longitudinal, 2 waves	N=4327<br><br>Grade 7-12<br><br>52% male & 48% female	Noncompletion of HS	Early smoking in grade 7-> School dropout	Substance use disorder (SUD)<br><br>3 groups of early smoking:<br>nonsmokers<br>experimenaters<br>smokers		Academic problems<br>Delinquency<br>Pregnancy	Compared with nonsmokers, smokers by grade 7 were 5 times more likely to drop out of school at grade 12.<br>Compared with experimenters, smokers by garde 7 were twice as likely to drop out	low

Reference	Study design	Sample<br>**N, age range, gender**	Dropout definition<br>**HS: High school**<br>**GED: General Educational Development**	Hypotheses	Disorder types	Evaluation of disorders	Confounding factors	Significant results<br>**ORadj: adjusted odds ratio**<br>**CI: confidence interval**<br>**RR: relative risk**	Overall risk of bias<br>**(for details, see additional file 4)**
Bray et al. [58]<br>2000<br>USA	longitudinal, 4 waves	N=1392 (at wave 4)<br><br>grades 6-11<br><br>51% male and 49% female	Non completion (dropout or expulsion) of HS at age 16<br><br>Noncompletion of HS at age 17<br><br>Noncompletion of HS at age 18	Marijuana initiation -> HS dropout	Substance use:<br>alcohol<br>cigarettes<br>marijuana<br>other illicit drugs (cocaine, crack, hallucinogens, stimulants, sedatives, inhalants)	Initiation of use prior to age 16, 17 or 18	Parents' education<br>Household composition<br>Race<br>Gender<br>Rural VS urban school<br>Grades earned	Marijuana initiation was significantly related to HS dropout for all ages, with an estimated association similar in magnitude and corresponding to an odds ratio of 2.3.<br><br>When controlling for initiation of multiple substance use, the association between marijuana use and HS dropout was significant for ages 16 and 18 only.<br>Alcohol use was not significantly related to HS dropout for all ages.<br>Cigarette initiation was significantly related for age 17 only.<br>The initiation of other drugs was insignificant in all models.	high
Hansen et al. [61]<br>1999<br>Pittsburgh, USA	case control study, 4 waves<br><br>case: diagnosis of Attention Deficit Disorder with hyperactivity (ADHD) between ages 7-17<br><br>control: no psychiatric illness	N=36 (18 cases & 18 controls)<br><br>18-26 years<br>mean: 20.4 for ADHD and 21.5 for controls)<br><br>**100% male**	HS incompletion with or without a GED	ADHD -> School dropout	At wave 1:<br>conduct disorder<br>simple phobia<br>major depression<br>bipolar disorder<br>enuresis<br>social phobia<br>Tourette's disorder<br>Adjustment disorder<br>Sleep disorder<br>Oppositional Defiant Disorder	Schedule for Affective Disorders and Schizophrenia for school-aged children (K-SADS)	not applied	Controls were more likely to have graduated from HS (89%) than ADHD subjects (56%) ($\chi 2=3.46$; p=0.63) and ADHD subjects were more likely to have attained a GED (28% $\chi 2=3.2$; p=0.088) than controls (6%).<br>No significant difference between cases and controls in occupational status.<br>ADHD subjects had more psychological difficulties in the past 5 years than controls ($\chi 2=6.84$; $p<0.01$)	high
Miech et al. [10]<br>1999<br>New Zealand	longitudinal, 9 waves<br><br>Dunedin Study	N=901<br><br>15-21 years (study based on waves 7 and 9)	Noncompletion of one of the educational milestones:<br>*No school certificate (=national exam at age 16)*<br>*School certificate*<br>*Earning a HS degree*<br>*Entering tertiary education*	Mental disorders -> Educational attainment	Anxiety disorder<br>Mood disorder<br>Antisocial disorder<br>Attention deficit disorder	Diagnostic Interview Schedule for Children (DISC)<br><br>Diagnostic Interview Schedule (DIS)	Socioeconomic status<br>Gender<br>IQ<br>Academic ability<br>School involvement	Internalizing disorders did not significantly affect educational attainment in any of the models.<br>In contrast, conduct disorders were significantly associated with failure to earn a HS degree (ORadj=3.35, p-value<0.01 for DSM disorder diagnosis and Oradj=1.49, p-value<0.01 for symptom assessment).<br>ADD was only significantly related to HS dropout when evaluated ina dimensional format.<br>A priori no gender differences and no influence related to comorbidity	low
Krohn et al. [49]<br>1997<br>Rochester,New York,USA	longitudinal, 10 waves<br><br>Rochester Youth Development Study	N=775<br><br>13-21 years<br><br>73% male & 27% female	Non completion of HS by age 21	Early substance use -> School dropout	Early substance use at wave 4 (alcohol, marijuana, crack, cocaine, hallucinogens, heroin, inhalants, tranquilizers, amphetamines, barbiturates).<br>Later alcohol use at wave 10<br>Later substance use at wave 10 (other illicit drugs)	Frequency of use across the first 4 waves	Peer use of alcohol & cannabis<br>Peer use of crack & hard drugs<br>Parental use of drugs & alcohol<br>Attachment to parents<br>Commitment to school<br>Social class/status<br>Race/ethnicity	After controlling for confounding factors, early substance use was significantly related to school dropout for males, but not for females.	low
Kessler et al. [5]<br>1995<br>USA	cross-sectional<br><br>National Comorbidity Survey (NCS)	N=5877<br><br>15-54 years	Noncompletion of one of the educational milestones:<br>*primary education*<br>*highschool*<br>*college entry*<br>*college graduation*	Psychiatric disorders -> Educational attainment	Mood disorder<br>Anxiety disorder<br>Conduct disorder<br>Substance use disorder (drug/alcohol abuse/dependence)	Composite International Diagnostic Interview (CIDI)	Age<br>Grade in school<br>Socioeconomic status<br>Being raised in a female-headed household<br>Number of siblings<br>Nativity<br>Urbanicity<br>Region of residence	All 4 types of disorders were significant predictors of failure in all 3 educational transitions:<br>*Noncompletion of HS:*<br>Anxiety: OR=1.4 CI=1.1-1.8; p<0.05<br>Mood: OR=1.5 CI=1.1-1.9; p<0.05<br>Substance use: OR=2.3 CI=1.8-3.1; p<0.05<br>Conduct: OR=2.4 CI=1.8-3.2; p<0.05<br><br>There was also a clear "dose-response" relationship between the number of prior disorders and dropping out of HS.<br><br>Conduct disorders were the most impeding among males for all 3 educational transitions whereas anxiety disorders<br><br>were the most impeding for females.	low
