# Supplementary material for: The downward spiral of mental disorders and educational attainment: a systematic review on early school leaving
Source: BMC Psychiatry. 2014 Aug 27;14:237. doi: 10.1186/s12888-014-0237-4 (PMC4244046; doi:10.1186/s12888-014-0237-4)
Supplement: Additional file 2: — Included references (school dropout as predictor of mental disorders). [file 12888_2014_237_MOESM2_ESM.pdf]

Additional file 2 Included references (school dropout as predictor of mental disorders)

Reference	Study design	Sample<br>**N, age range, gender**	Dropout definition<br>**HS: High school**<br>**GED: General Educational Development**	Hypotheses	Disorder types	Evaluation of disorders	Confounding factors	Significant results<br>**ORadj: adjusted odds ratio**<br>**CI: confidence interval**<br>**RR: relative risk**	Overall risk of bias<br>**(for details, see additional file 4)**
Apantaku-Olajide et al.<br>2014<br>Dublin, Ireland	cross-sectional	N=193 (63 school dropouts, 46 students in alternative education, 84 mainstream students)<br><br>mean age: 16.1 years	Having left secondary school	School dropout -> substance use<br><br>Prior mental disorder -> school dropout	Past month and lifetime substance use<br><br>Attention Deficit Disorder<br>Conduct disorder<br>Depression<br>Anxiety disorder<br>Psychosis	Self-report for substance use<br><br>Clinical evaluation for mental disorders	not applied	Compared with mainstream students, droputs reported more parental substance use OR=4.4 (95% CI: 1.9-10.4), p-value< 0.007 and more polysubstance use.<br>Earlier age of substance abuse onset among dropouts.<br>Having a prior mental disorder was significantly associated with school dropout OR=2.7 (95% CI: 1.3-5.5), p-value<0.007.	unclear
Ohayon & Weiss<br>2014<br>USA	cross-sectional	N=2082<br><br>18-26 years, mean 21.2 years	Non completion of HS	School dropout -> depressive mood, major depressive disorder	Depressive mood<br>Major depressive disorder	Symtom criteria of DSM IV	Gender<br>Age<br>Weight	23.5% of youth reported depressive mood (95%CI: 21.4-25.2%)<br>Prevalence was comparable between students, working and not working youth. However for working youth, having dropped out of HS was significantly related with depressive mood OR=2.9 (95%CI: 1.3-6.5), p-value<0.01.	unclear
Benjet et al.<br>2012<br>Mexico City, Mexico	cross-sectional<br><br>Mexican Adolescent Mental Health Survey	N=3005<br><br>12-17 years, mean 21.2 years	Non completion of HS<br>NEET (Not in Education, Employment or Training)	School dropout -> psychiatric disorder	Mood disorders<br>Anxiety disorders<br>Substance use disorders<br>Behavioural disorders<br>Suicidal behaviour	Composite International Diagnostic Interview (CIDI)	Gender<br>Age<br>Socioeconomic status<br>Household composition	Except for anxiety, NEETs had higher odds for psychiatric disorders than students, even after adjustment:<br>ORadj for mood disorders=2.7 (95%CI: 1.8-4.2), p<0.0001<br>ORadj for substance use disorder=4.4 (2.6-7-6), p<0.0001<br>ORadj for behavioural disorders=2.1 (1.5-2.8), p<0.0001<br>ORadj for any disorder=1.7 (95%CI: 1.3-2.3), p<0.0001<br>They also reported significantly higher odds for suicidal behaviour.	low
Lee et al. [50]<br>2010<br>Minnesota, USA	longitudinal, 3 waves<br><br>Minnesota Competence Enhancement Project	N=310<br><br>76% male & 24% female	Non completion of HS	High School dropout -><br>Substance use disorder	Substance use disorder	Diagnostic Interview for Children and adolescents (DICA-R)	Early onset of substance use<br>History of using illicit drugs<br>Parental history of drug problems<br>Gender<br>Childhood attention deficit disorder, conduct disorder and oppositional defiant disorder	Definition of 2 different classes:<br>1. **very low rate class**: proba of being diagnosed with abuse or dependence at T1, T2 and T3 were less than 0.13 for alcohol and less than 0.05 for cannabis.<br>2. **moderate rate class**: proba of being diagnosed with abuse or dependence at T1, T2 and T3 was between 0.21 and 0.49 for alcohol and between 0.22 and 0.55 for cannabis.<br>HS dropouts were more likely to be in the moderate rate class for cannabis use than those who complete college (ORadj=18.62 CI=2.24-154.90; p<0.05). There was no significant association between HS dropout and moderate alcohol use.	unclear
Benjet et al. [25]<br>2009<br>Mexico City, Mexico	cross-sectional<br><br>Mexican Adolescent Mental Health Survey	N=3005<br><br>12-17 years	Noncompletion of junior HS or HS	School dropout -> Mental disorder	Anxiety disorders<br>Mood disorders<br>Impulse control disorder<br>Substance use disorder (SUD)	Composite International Diagnostic Interview (CIDI)	Age<br>Gender<br>Family constellation<br>Adolescent burden (work, married or having children)<br>Parental education	Compared to students, school dropouts were significantly more likely to have any mental disorder (ORadj=1.51 CI=1.15-1.98; p<0.05), a mood disorder (ORadj=2.16 CI=1.49-3.13; p<0.05), any impulse control disorder (Oradj=1.44 CI=1.09-1.91; p<0.05) or to have a SUD (ORadj=2.16 CI=1.32-3.52; p<0.05). But there was no difference between students and dropouts in the prevalence of anxiety disorders.	unclear
Crosnoe & Riegle-Crumb [53]<br>2007<br>USA	longitudinal, 3 waves	N=6038<br><br>Grades 9-12<br><br>48% male & 52% female	Noncompletion of HS<br>Noncompletion of 2-year college<br>Noncompletion of 4-year college	Educational status -> Alcohol use<br>High academic status in HS -> College -> Social drinking<br>Low academic status or Dropout in HS -> Labor -> Less drinking opportunities	Alcohol dependence/abuse	Frequency of drinking in the past year<br><br>Frequency of binge drinking in the past years	Gender<br>Race/ethnicity<br>Parent education<br>Grade level as a proxy for age<br>Family structure<br>Athletic status	Participants who continued their academic career after HS were more likely to become current drinkers than their peers who finished their academic careers with HS or who dropped out.	low
Fothergill et al. [35]<br>2007<br>Chicago, USA	longitudinal, 4 waves<br><br>Woodlawn Study	N=1048<br><br>6-42 years<br>48% male & 52% female<br><br>**Urban African Americans**	Noncompletion of HS<br>Having a GED<br>Noncompletion of college	Poor educational achievement -><br>Drug use disorders	Substance use disorder	Composite International Diagnostic Interview (CIDI)	Gender<br>Mother's self-report of drug use	After adjustment for gender and mother's self-report of drug or alcohol use, HS dropouts were significantly more likely to report drug use disorders (ORadj=3.5 CI=1.71-7.17; p<0,001) compared to those with a college degree.<br>Having a HS degree, a GED or training reduces this OR to 2.63 (CI=1.34-5.17; p=0,04).<br>Poor school engagement (skipping classes & suspensions) were also related to later drug use disorders	unclear

Reference	Study design	Sample<br>N, age range, gender	Dropout definition<br>HS: High school<br>GED: General Educational Development	Hypotheses	Disorder types	Evaluation of disorders	Confounding factors	Significant results<br>ORadj: adjusted odds ratio<br>CI: confidence interval<br>RR: relative risk	Overall risk of bias<br>(for details, see additional file 4)
Williams et al. [55]<br>2006<br>Ontario, Canada	cross-sectional	N=2070<br>**clinical sample: childhood adversities**<br><br>19-64 years (mean: 39.3 years for men & 37.8 years for women)<br><br>55% male & 45% female	Noncompletion of secondary school (grade 12)	Childhood adversities -> Educational attainment -> Mental health	Anxiety disorder<br>Depressive disorder<br>Substance use disorder<br>Antisocial behavior	Child Maltreatment History Self-report (CMHSR)<br><br>Composite International Diagnostic Interview (CIDI)	Socioeconomic status<br>Age<br>Gender<br><br>Academic achievement	Lower acheivers who did not complete HS had significantly higher odds (ORadj=1.55 CI=1.00-2.38; p=0.048) for experiencing internalizing disorders compard to their higher achieving counterparts.<br>For both, higher and lower achievers, dropping out of HS was significantly related to experiencing externalizing disorders (ORadj=1.69 CI=1.02-2.80; p=0.043 and ORadj=1.93 CI=1.14-3.30; p=0.015 respectively).<br>In general, HS completion, indpendent of higher achievement status, was associated with lower rates of externalizing but not internalizing disorders.	unclear
Harford et al. [36]<br>2006<br>USA	longitudinal, 10 waves<br><br>National Longitudinal Survey of Labor Market Experience in Youth	N=8661<br><br>19-25 years at wave 1 in 1984<br><br>51% male & 49% female	Noncompletion of HS<br>Graduation of HS (reference)<br>1 year of college<br>2 years of college<br>3 years of college<br>more than 4 years of college	School dropout -> Alcohol use disorder 10 years later	Alcohol use disorder (AUD)		Age<br>Gender<br>Race<br>Poverty status at wave 1<br>Martital status at wave 10<br>Family history of alcohol use<br>Age at onset of alcohol use<br>Antisocial behaviors<br>Illicit substance use	Educational status at wave 1 significantly predicted AUD at wave 10. The risk for alcohol dependence at wave 10 was highest among those who dropped out of HS (ORadj=1.57 CI=1.24-1.98; p<0.01), even after controlling for other background and behavioral predictors.<br>Compared to HS graduates, HS dropouts had a slightly but significantly lower risk for alcohol abuse (ORadj=0.81 CI=0.67-0.99; p<0.05).<br>Thus, increasing educational attainment was associated with decreased likelihood of reporting alcohol dependence but not alcohol abuse at longterm follow-up.	unclear
Daniel et al. [46]<br>2006<br>USA	longitudinal, 4 waves	N=188<br><br>15.0-15.9 years; mean: 15.4 years<br><br>56% male & 44% female	Noncompletion of HS<br>Pursuing a GED<br>Receiving vocational training<br>Incarceration	School dropout -> Suicidality	Major depressive disorder<br>Conduct disorder/Oppositional defiant disorder<br>Substance use<br>Suicidal behaviors	Schedule for Affective Disorders and Schizophrenia for school-aged children (K-SADS)	Age<br>Gender<br>Socioeconomic status<br>Race/ethnicity<br>Psychiatric morbidity	School dropout was related to an increased incidence of suicidality (ORadj=11.03; p<0.001)	unclear
Kogan et al. [52]<br>2005<br>USA	longitudinal, 2 waves<br><br>Data from the National Educational Longitudinal Study	N=1762<br><br>grade 8-12<br><br>49% male & 51% female<br><br>**African American youth**	Noncompletion of HS	School dropout -> Substance use	Alcohol use disorder<br>Marijuanan use<br>Cigarette use	Frequency of use during the past 30 days for alcohol and cannabis<br><br>Daily use for cigarettes	Gender<br>Parenthood status<br>Socioeconomic status<br>Time since dropout<br>Behavior problems in school<br>Academic problems	18% of the participants dropped out of school.<br>School dropouts were 2.2 times more likely to report cigarette use and 1.3 times more likely to report marijuana use during the past 30 days than were students. These effects were independent of prior problem behaviour and substance use. There was no significant link with alcohol.	low
D'Amico et al. [34]<br>2005<br>California, Oregon, USA	longitudinal, 3 waves	N=1986<br><br>51% male & 49% female<br><br>Mean age (wave 1)=18.13 years<br>Mean age (wave 2)=23.50 years<br>Mean age (wave 3)=29.44 years	Non completion of HS by age 18	School dropout -> Substance abuse/ dependence	Deviance<br>Substance use disorder<br>Hard drug use<br>Mental health status	Mental Health Inventory-5 (MHI-5)<br>DSM IV criteria for abuse or dependence<br>but not based on a diagnostic interview	Gender<br>Race/ethnicity<br>Age at wave 1<br>Family history of substance use<br><br>Occupation status<br>Educational attainment<br>Family composition<br>Pro-drug social influences	School dropout at age 18 was associated to drug dependence but not abuse, in univariate analysis only.<br>If entered in the multivariate models, school dropout was not significantly related to drug dependence nor abuse.	low
Fergusson et al. [42]<br>2003<br>Christchurch, New Zealand	Birth cohort, 21 waves<br><br>Christchurch Health and Development Study	N=1265<br><br>15-25 years<br><br>50% male & 50% female	HS Dropout: having left school by age 18 without achieving at least one pass grade in School Certificate examinations	High School dropout -> Cannabis use		Frequency of cannabis use for a 12-month period	Socio-demographic factors<br>Changes of parents<br>Childhood adversities<br>Parental attachment at 15 years<br>Parental adjustment<br>Childhood cognitive ability<br>Gender<br>Cigarette smoking<br>Novelty seeking<br>Deviant peer affiliations<br>Childhood behavior problems<br>Conduct problems	*School dropout -> subsequent cannabis use:*<br>After adjustment for sign. confounding factors, there was no detectable association between educational achievement and subsequent cannabis use.	low

Reference	Study design	Sample<br>**N, age range, gender**	Dropout definition<br>**HS: High school**<br>**GED: General Educational Development**	Hypotheses	Disorder types	Evaluation of disorders	Confounding factors	Significant results<br>**ORadj: adjusted odds ratio**<br>**CI: confidence interval**<br>**RR: relative risk**	Overall risk of bias<br>**(for details, see additional file 4)**
Liem et al. [54]<br>2001<br>Boston, USA	longitudinal, 2 waves<br><br>comparison study: HS graduates	N(graduates)=1143<br>>= 16 years<br>48% male & 52% female<br><br>N(dropouts)=182<br>>=18<br>52% male & 48% female	Non completion of HS	High School dropout -> Mental health difficulties	Depression<br>Anxiety<br>Self-esteem	Cutrona'a Social Provisions Scale<br>Collin's & Read's attachment instrument<br>Center for Epidemiological Studies Depression Scale (CES-D)<br>Symptom Checklist-90<br>Rosenberg's Self-esteem Scale	Household composition<br>Parental support<br>Transition specific career help<br>Openness to adult support<br>Mental health status at T1<br><br>Gender<br>Race<br>Family socioeconomic status	Dropouts reported significantly more depression than graduates at both times of follow-up. However, at T1, this difference was significant for dropouts with low parental support only, but not for dropouts with high parental support.<br><br>Adding depression at T1 as a control, rendered the effect of dropout on depression at T2 insignificant. They did not report more anxiety at the moment of graduation, but 2 years later, at T2, they did. There was no significant difference between dropouts ad graduates in self-esteem, at either T1 or T2.	unclear
Obot et Anthony [38]<br>2000<br>USA	case control study<br><br>case: injecting drug use (IDU)<br>control: non-IDU<br>matching: neighborhood & year of assessment<br><br>Data from the NHSDA	N=1401 (226 IDUs & 1175 non-IDUs)<br>**Non-Hispanic White Americans**<br><br>>= 18 years, mean: 32.3 years for IDUs & 33.1 for non-IDUs<br><br>IDUs: 138 male & 88 female<br>Non-IDUs: 482 male & 693 female	Non attendance of HS<br><br>Non completion of HS without GED<br><br>Non completion of HS with GED<br><br>HS graduation<br><br>Non completion of college	Educational attainment -> Injecting Drug Use (IDU)<br>(lifetime, past & recent)	Injecting Drug Use (IDU):<br>Lifetime IDU<br>Recent IDU<br>Past IDU		Age<br>Gender	Compared with HS graduates, dropouts without a GED were more likely to have started injecting drugs ORadj= 2.2 CI=1.31-3.60; p=0.003). This association was even stronger for dropouts with a GED (ORadj=3.4 CI=1.99-5.71; p<0.001).<br>HS dropouts with a GED were 4 times more likely te be recent drug injectors (ORadj=4.4 CI=1.31-14.96; p=0.017) whereas the association between HS dropout without GED and injecting drug use was not significant.<br>Being a HS dropout with or without GED was associated with having been an IDU in the past (ORadj=3.4 CI=1.81-6.50; p<0.001 and ORadj=2.5 CI=1.36-4.61; p=0.003 respectively).	unclear
Obot et al. [39]<br>1999<br>USA	case control study<br><br>case: injecting drug use (IDU)<br>control: non-IDU<br>matching: neighborhood & year of assessment<br><br>Data from the National Household Survey on Drug Abuse (NHSDA)	N=2642 (389 IDUs & 2253 non-IDUs)<br>**African Americans**<br><br>>= 18 years, mean: 34.1 years for IDUs & 33.3 for non-IDUs<br><br>IDUs: 229 male & 160 female	Non attendance of HS<br><br>Non completion of HS without GED<br><br>Non completion of HS with GED<br><br>HS graduation	Educational attainment -> Lifetime Injecting Drug Use (IDU)	Lifetime injecting drug use (IDU)		Age<br>Gender<br>Hispanic background	Compared with HS graduates, dropouts without a GED were more likely to have started injecting drugs (ORadj= 1.9 CI=1.3-2.6; p<0.001). This association was even stronger for dropouts with a GED (ORadj=2.3 CI=1.4-3.8; p<0.001).	unclear
Obot et Anthony [37]<br>1999 (b)<br>USA	case control study<br><br>case: injecting drug use (IDU)<br>control: non-IDU<br>matching: neighborhood & year of assessment<br><br>Data from the NHSDA	N=2579 (226 IDUs & 2353 non-IDUs)<br>**African Americans**<br><br>>= 18 years, mean recent IDU: 30,7 years, mean non-IDU: 33.5 mean past IDU: 37.3 years, mean non-IDU: 33.9<br><br>IDUs: 129 male & 97 female<br>Non-IDUs: 859 male & 1494 female	Non attendance of HS<br><br>Non completion of HS without GED<br><br>Non completion of HS with GED<br><br>HS graduation<br><br>Non completion of college	Educational attainment -> Recent and past Injecting Drug Use (IDU)	Recent and past injecting drug use (IDU)		Age<br>Gender<br>Hispanic background	Compared with HS graduates, dropouts without a GED were more likely to have had recent IDU (ORadj=2.2 CI=1.25-4.02; p=0.007). This association was even stronger for dropouts with a GED (ORadj=2,7 CI=1.18-6.25; p=0.018).<br><br>Compared with HS graduates, GED holders were over-represented among past IDUs (ORadj=3.1 CI=1.19-8.27; p=0.021). There was no significant association of any other educational category and past IDU.	unclear
Miech et al. [10]<br>1999<br>New Zealand	longitudinal, 9 waves<br><br>Dunedin Study	N=901<br><br>15-21 years (study based on waves 7 and 9)	Noncompletion of one of the educational milestones:<br>*No school certificate (=national exam at age 16)*<br>*School certificate*<br>*Earning a HS degree*<br>*Entering tertiary education*	Educational attainment->Mental disorders	Anxiety disorder<br>Mood disorder<br>Antisocial disorder<br>Attention deficit disorder	Diagnostic Interview Schedule for Children (DISC)<br><br>Diagnostic Interview Schedule (DIS)	Family socioeconomic status<br>Gender<br>Age 15 disorder	Lower educational attainment was significantly associated with increases in anxiety and antisocial disorders but not depression. Monotonic relationship.<br>The effects of low educational attainment on antisocial disorders may be greater for men than women.	low
Crum et al. [60]<br>1998<br>Chicago, USA	longitudinal, 3 waves<br><br>Woodlawn Study	N=953<br><br>48% male & 52% female<br><br>**Urban African Americans**	Non completion of grade school<br>Non completion of HS<br>Having a GED<br>Graduating from HS<br>Non completion of college	School dropout -> Alcohol abuse and dependence in adulthood	Alcohol use disorder	Composite International Diagnostic Interview (CIDI)	Gender	Dropping out of HS was associated with a two-fold greater risk of an alcohol use disorder (RR adjusted for sex: 2.1 CI=1.0-4.4; p<=0.05).<br>There was no statistically significant association of any other educational category with alcohol abuse.<br>After removing from the sample individuals with a report of early onset problem drinking, the results were similar, except for people with a GED whose risk decreased.<br>Finally, predictors of alcoholism included teacher's rating of underachievement in first grade, few family rules about school, infrequent homework with family, low educational aspirations and HS dropout.	high

Reference	Study design	Sample<br>**N, age range, gender**	Dropout definition<br>**HS: High school<br>GED: General Educational Development**	Hypotheses	Disorder types	Evaluation of disorders	Confounding factors	Significant results<br>**ORadj: adjusted odds ratio<br>CI: confidence interval<br>RR: relative risk**	Overall risk of bias<br>**(for details, see additional file 4)**
Gfroerer et al. [51]<br>1997<br>USA	cross-sectional<br><br>Data from the National Household Survey on Drug Abuse (NHSDA)	N=12026<br><br>17-22 years	Non completion of HS	HS dropout -> Substance use<br><br>College enrollment -> Substance use as students are living away from their parents	Alcohol use<br>Substance use (marijuana, cocaine, cigarettes)	past month substance use<br>heavy alcohol use: having 5 or more drinks per occasion on 5 or more different days in the past month	Age<br>Race<br>Gender	Compared to HS graduates, dropouts were more likely to have used marijuana in the past month (ORadj=1.36 CI=1.06-1.76; p=0.02) and to be current smokers (ORadj=2.59 CI=2.17-3.08; p<0.01) whereas they were less likely to have used alcohol (ORadj=0.84 CI=0.69-1.02; p=0.07).<br><br>Compared to those living with parents, college students living without parents were 2 times more lielky to report heavy alcohol use (ORadj=2.25 CI=1.49-3.39; p<0.01).	unclear
Krohn et al. [49]<br>1997<br>Rochester,New York,USA	longitudinal, 10 waves<br><br>Rochester Youth Development Study	N=775<br><br>13-21 years<br><br>73% male & 27% female	Non completion of HS by age 21	School dropout -> Substance use in young adulthood	Early substance use at wave 4 (alcohol, marijuana, crack, hallucinogens, heroin, inhalants, tranquilizers, amphetamines, barbiturates, cocaine).<br>Later alcohol use at wave 10<br>Later substance use at wave 10 (other illicit drugs)	Frequency of use across the first 4 waves	Peer use of alcohol & marijuana<br>Peer use of crack & hard drugs<br>Parental use of drugs and alcohol<br>Attachment to parents<br>Commitment to school<br>Social class/status<br>Race/ethnicity	For females, school dropout increased the probability of later drug use, but did not affect alcohol use. For males, school dropout increased the probability for both, alcohol and drug use.	low
Tresidder et al. [59]<br>1997<br>New South Wales, Australia	cross-sectional	N=283<br><br>16-year-old school dropouts registered with the Common-Wealth Employment Service between June-November 1994<br><br>61% male & 39% female	Having left school before or at the end of year 10	School dropout -> Mental health & risk behaviour	Tobacco<br>Alcohol use<br>Cannabis use	NSW Schools Drug and Alcohol Survey	Gender<br>Method of recruitment	Among school dropouts, gender was not associated with cigarette or marijuana use, but boys were more likely than girls to report binge drinking (67% VS 34%; p=0.007)<br><br>When compared to an in-school sample, dropouts had significantly higher rates of weekly tobacco and cannabis use, for both boys and girls. Higher rates of binge drinking were observed for male dropouts only.	high
