# Supplementary material for: The downward spiral of mental disorders and educational attainment: a systematic review on early school leaving
Source: BMC Psychiatry. 2014 Aug 27;14:237. doi: 10.1186/s12888-014-0237-4 (PMC4244046; doi:10.1186/s12888-014-0237-4)
Supplement: Additional file 3: — References without DSM criteria - Addendum. [file 12888_2014_237_MOESM3_ESM.pdf]

Additional file 3 References without DSM criteria - Addendum

Reference	Study design	Sample<br>**N, age range, gender**	Dropout definition<br>**HS: High school**<br>**GED: General Educational Development**	Hypotheses	Disorder types	Evaluation of disorders	Confounding factors	Significant results<br>**ORadj: adjusted odds ratio**<br>**CI: confidence interval**<br>**RR: relative risk**	Limitations
Pingault et al.<br>2011<br>Québec, Canada	longitudinal, 7 waves	N=2000<br><br>6-23 years<br><br>1001 male & 999 female	Noncompletion of secondary education	Attention Deficit Hyperactivity Disorder -> educational attainment	Attention Deficit Hyperactivity Disorder (ADHD)	Social Behavior Questionnaire	Gender<br>Birth weight<br>Socioeconomic variables<br>Family structure<br>Parental separation<br>Residential move	46.3% had constantly low symptoms of inattention, whereas 16.8% had constantly high symptoms. For 17.6% symtoms were rising whereas for 19.3% they were declining. Compared with the low symptoms trajectory, the declining trajectory (ORadj=2.67, 95%CI: 1.90-3.75), the rising trajectory (ORadj=3.87, 95%CI: 2.75-5.45) and high inattention group (ORadj=7.66, 95%CI: 5.06-11.58). In contrast, hyperactivity was not significantly related to school dropout when inattention was controlled.	Teacher-report only for symptom assessment.
Gagné et al. [68]<br>2011<br>Québec, Canada	longitudinal	N=537<br><br>Mean age at t1: 13 years<br>Mean age at t2: 20 years<br><br>52% male & 48% female	Not having obtained the the diploma of secondary education and not being registered with a school by the age of 20	Depression -> School experiences -> Dropout	Depressive symptoms	BDI<br><br>Echelle de l'environnement de classe	Academic results<br>Social affiliation<br>Relationship with teacher<br>Commitment	No significant difference was detected between depressed and non-depressed students regarding their educational attainment:12.6% of depressed students had dropped out of school, compared to 11.2% of non-depressed students.<br>School dropouts had higher scores of depression than students. This difference was even stronger for girls.<br>Depressed students having poor academic results were 5 times more likely to drop out of school than non-depressed students.	Small sample of students diagnosed with a depressive episode<br><br>The control group of students may included former dropouts who reregistered with school
Frensch et al.<br>2009<br>Ontario, Canada	longitudinal, 3 waves<br><br>**comparison between 2 clinical groups:**<br>RT: youth in residential treatment<br>IFS: youth in intensive family services	N(RT)=106 caregivers & 33 youth<br><br>N(IFS)=104 caregivers & 35 youth<br><br>Average age at admission: 11.55 years<br>Average age at follow-up: 14.11 years (RT); 13.65 years (IFS)<br><br>79% male & 21% female (RT)<br>71% male & 29% female (IFS)	Non completion of secondary school		Role performance<br>Delinquency<br>Behavior towards others<br>Mood/emotions (depression & anxiety)<br>Self-harm behavior<br>Substance use<br>Problems in thinking	Child and Adolescent Functional Assessment Scale (CAFAS)		At admission, there were significantly more IFS youth in school than RT youth (95.2% versus 87.6%).<br>At follow-up, among youth 16 or older, the percentages not in school increased to 54.1% for RT youth and 31.6% for IFS youth.<br>Among RT school dropouts, 71% left school by choice, whereas among IFS youth, 53.4% were suspended or expulsed.<br>School dropout was best predicted by juvenile delinquency: for every unit increase in impairment severity, youth were almost 4 times more likely to drop out.	The predictive model of school dropout should be considered with caution as only 35.7% of the sample were over the age of 16 at follow-up.
Trampush et al. [69]<br>2008<br>USA	case control study<br><br>case: ADHD combined type diagnosis in childhood<br><br>control: no ADHD diagnosis<br><br>matching: gender, ethnicity, socio-economic status	N=93<br><br>16-21 years (mean: 18.81 years)<br><br>91% male & 9% female	Noncompletion of HS, pursuing or having completed a GED	ADHD diagnosis in childhood -> HS dropout	ADHD combined type<br><br>Polysubstance use (marijuana, cocaine, amphetamine, morphine)	Diagnostic Interview Schedule for Children (DISC)<br><br>CBCL<br>Conner's rating scale<br><br>Rutgers Drug and Alcohol Questionnaire (RDAQ) & urinalysis	General cognitive functioning<br>Reading ability<br>Contact with biological father	Cognitive functioning (OR=7.3; p<0.001), substance use (OR=3.2; p=0.002) and amount of contact with father (OR=2.4; p<0.01) were significantly related to school dropout.<br>But, none of the factors differentially influenced the risk of dropout in adolescents with ADHD compared to adolescents without ADHD.	Design and sample size did not allow to compare dropouts rates between ADHD adolescents and controls<br><br>Title and content of the study did not match…<br><br>Combined measures for substance use (self-report & physiological)
Ou<br>2008<br>Chicago, US	longitudinal<br><br>Chicago Longitudinal Study (CLS)	N=1372<br><br>0-24 years<br><br>48% male & 52% female<br><br>**Low income minority youth**	Noncompletion of HS<br>Having a GED<br>Graduating from HS	Educational attainment -> Health outcomes<br><br>HS graduates are associated with better outcomes than GED recipients, and GED recipients are associated with better out-comes than HS dropouts	Depressive disorder<br>Substance use (alcohol, marijuanan, other illicit drugs)	Derogatis Brief Symptom Inventory, Depression Subscale	Family SES<br>Supportive program particpation<br><br>Early scholastic skills	HS dropouts had significantly more symptoms of depression than GED recipients (marginal effect=9.5% p=0.02). Except for severe depressive symptoms, there was no significant difference between GED recipients and HS graduates.<br>Compared to GED recipients, dropouts reported significantly more substance use since age 16 (marginal effect=13.1%; p<0.005) whereas HS graduates reported significantly less use (marginal effect= -11.8%; p=0.001). Regarding severe substance use, there was no significant difference between GED recipients and HS dropouts.	Correlational results, not causal interpretations<br><br>Specific sample characteristics<br><br>Self report data
Bond et al.<br>2007<br>Victoria, Australia	longitudinal, 3 waves	N=2400<br><br>14-17 years<br><br>47% male & 53% female	Noncompletion of year 12 (last year of secondary school)	Social and school connectedness -> Mental health -> Educational attainment	Overall mental health status<br>Substance use	Clinical Interview Schedule Revised (CIS-R)	Social connectedness<br>Interpersonal conflict<br>School connectedness<br>Family structure<br>Parental level of education<br>Language spoken at home	Smoking in year 8 (ORadj=0.43 CI=0.21-0.91) and anxious/depressive symptoms (ORadj=0.68 CI=0.41-1.13) decreased the odds of completing school whereas marijuana and alcohol had no significant association with school completion.<br>Year 8 students with very low school connectedness were more likely to report depressive symptoms and engage in substance use in year 10, and less likely to finish school at all.	Attrition rate of wave 3

Reference	Study design	Sample<br>**N, age range, gender**	Dropout definition<br>**HS: High school**<br>**GED: General Educational Development**	Hypotheses	Disorder types	Evaluation of disorders	Confounding factors	Significant results<br>**ORadj: adjusted odds ratio**<br>**CI: confidence interval**<br>**RR: relative risk**	Limitations
Reschly & Christenson[14]<br>2006<br>USA	longitudinal, 2 waves<br><br>Data from the National Educational Longitudinal Study (NELS)	N=1064 students identified with learning difficulties (LD)<br>N=338 students identified with emtional/behavioral disorders (EBD)<br>N=96 students identified with LD & EBD<br><br>**Youth with LD and/or EBD**	Non completion of secondary school at wave 2	Engagement (Behavioural, cognitive and psychological) -> School dropout	EBD<br>LD	NCES definitions	SES<br>Grade retention<br>Academic performance	Among students with LD, 26% dropped out of school. Among students with EBD, almost half (49.9%) dropped out, compared to only 14.8% among students without any disability.<br>Among students with EBD, each covariate was a significant predictor of dropout.The strongest behavioural engagement predictor was cutting classes: the odds of dropping out were 3 times greater forevery unit increase.<br><br>Unexpectedly, higher perceptions of utility of education were associated with an increase in the odds of dropping out. Among students with LD, low SES and grade retention were predictors of school dropout. Idem EBD for cutting classes, whereas higher perceptions of utility decreased the odds. Covariates alone correctly classified a higher number of students without any disability than students with EBD or LD and adding the engagement variables did not improve the classification of students and dropouts among those without disabilities.	Extant database: measures were limited by the design and scope of the study<br><br>No measures of peer and teacher relationships<br><br>Selection of students with disabilities: variations by state, overlapping in the skill deficits among categories<br><br>Used data were dated
Quiroga et al.<br>2006<br>Canada	longitudianl, 3 waves	N=139<br><br>12-18 years (mean: 15.1; SD:1.7)<br><br>57% male & 43% female	Noncompletion of secondary school	Depressive symptoms -> School Dropout	Depressive symptoms	BDI	Gender<br>Age<br>Cognitive skills<br>SES<br>School experience	Depressive symptoms are significantly related to dropout: ORadjusted=1.66.<br>This association varied with gender (p=0.07), with a slightly stronger impact of depressive symptoms on educational attainment among boys (33% who dropped out against 31% among girls).	Small sample size<br><br>Selection bias (youth with a low SES were overrepresented)<br><br>Self-report data for depressive symptoms
Farahati et al.<br>2003<br>USA	cross-sectional<br><br>NCS	N=3389<br><br>19-54 years<br><br>48% male & 52% female	Non completion of HS	Parental metal illness -> School dropout among their children	*Parental disorders:*<br>Major depression<br>Anxiety disorder<br>Alcohol abuse<br>Drug dependence/abuse<br><br>*Youth disorder:*<br>Depressive disorders<br>Anxiety disorders<br>Alcohol dependence/abuse<br>Drug dependence/abuse<br>Conduct disorder		Gender<br>Age<br>Health status<br>Race<br>Religion<br>Native language<br>Teenage marriage/parenting<br>Family composition<br>Youth disorder<br>Parental education<br>Moves of family<br>Region of residence<br>Type of region<br>National unemployment rate	When controlling for confounding factors and comorbidities, maternal depression increased school dropout among their daugthers. Also, girls whose mothers have comorbid anxiety and alcohol disorders had a higher probability of dropout than girls whose mothers did not have those disorders.<br><br>Interestingly,a father's depression decreased the probability of dropout among his daughters. For men, no significant effects of parental mental illness on dropout were observed in the fullest model.<br>Reporting a psychiatric disorder prior to age 18 significantly increased the probability of dropout for both men and women.	Self-report/ -appreciation of parental mental illness -> bias in recognizing more "silent" disorder types
Hammarström & Janlert<br>2002<br>Sweden	longitudinal, 4 waves	N=1044<br><br>16-30 years<br><br>52% male & 48% female<br><br>**clinical: school leavers**	Non completion of secondary school	School dropout -> Early unemployment (>= 6 months between ages 16-21) -> Psychiatric disorders	Alcohol consumption<br>Psychological symptoms<br>Daily Smoking<br>Somatic symptoms		Length of unemployment<br>Working class background<br>Health behavior at age 16	School dropouts in early unemployment were significantly more likely to be daily smokers at age 30 than their working peers (ORadj= 3.5 CI=2.0-6.2) for men and for women (ORadj=2.4 CI=1.3-4.3). They also were more likely to to have psychological problems (ORadj=2.6 CI= 1.4-4.7 for men and ORadj=1.9 CI=1.0-3.5 for women respectively) and somatic symptoms (ORadj=1.7 CI=1.0 - 3.0 for men).	Arbitrary definition of cut-off point regarding alcohol consumption<br>The early unemployment group was defined irrespective of later employment -> possible exposure bias
Scanlon et Mellard<br>2002<br>Midwest, US	cross-sectional	4 groups considered:<br>1) HS dropouts with LD/EBD who obtained a GED: N=14<br>mean age: 19.2 years<br>2) HS dropouts without LD/EBD who are in adult education: N=90<br>mean age: 19.13 years<br>3) HS dropouts with LD/EBD who are in adult education: N=52<br>mean age: 18.28 years<br>4) HS students with LD/EBD:	Noncompletion of HS	Learning difficulties -> Dropout<br>Emotional or behavioral disorder-> School dropout	Learning difficulties (LD)<br>Emotional or behavioral disorder (EBD)	Self-report or school records	Educational history<br>School leaving<br>Post-leaving educational and economic experiences<br>Gender<br>Ethnicity<br>Use of special education services	In each of the 3 groups of school dropouts, 71% or more dropped out before completing 11th grade.<br>All 3 dropout groups more frequently reported behaviour or emotional disability-related problems than academic or physical disability problems.<br>However, 1/3 or more of the disability-related samples reported no disability-based influence on their daily activities.<br>More than 90% of participants with disabilities reported having used special education services with the lowest percentage for adult education enrollees.	Due to a small sample size:<br>Representativeness of the samples<br>Results limited to desriptive statistics<br><br>Self report data
Thornberry et al.<br>2001<br>Rochester,New York,USA	longitudinal, 10 waves<br><br>Rochester Youth Development Study	N=738 adolescents and caregivers<br><br>Mean age at wave 2: 14.5 years<br>Mean age at wave 9: 17.9 years<br><br>73% male & 27% female	Noncompletion of HS by age 20.1 years (wave 10)	Childhood and adolescent maltreatment -> HS dropout	Early childhood maltreatment<br>Late childhood maltreatment<br>Adolescence maltreatment<br>Persistent maltreatment		Gender<br>SES<br>Family composition<br>Race/ethnicity<br>Parental education<br>Community poverty	23% of the sample had dropped out of HS.<br>However, considering the timing of maltreatment, only late childhood maltreatement increased the odds for dropout by 1.81; whereas there was no association observed for early childhood-only maltreatement, adolescence-only maltreatment and persistent maltreatment. All analyses adjusted.	No consideration of PTSD

Reference	Study design	Sample<br>**N, age range, gender**	Dropout definition<br>**HS: High school**<br>**GED: General Educational Development**	Hypotheses	Disorder types	Evaluation of disorders	Confounding factors	Significant results<br>**ORadj: adjusted odds ratio**<br>**CI: confidence interval**<br>**RR: relative risk**	Limitations
James & Lawlor<br>2001<br>Ireland	cross-sectional	N=78<br><br>15-18 years<br><br>40 males & 38 females	Having left school without qualifications<br>Not having received or completed vocational training<br>Being at least 6 month in the labou market<br>Not being enrolled in full time education	School dropout -> Psychological problems	Withdrawn<br>Somatic complaints<br>Anxiety/depression<br>*togethter providing an internalizing score*<br>Social problems<br>Thought problems<br>Attention problems<br>Delinquency<br>Aggression<br>*together providing an externalizing score*<br><br>Thoughts of suicide and self-harm	Youth Self Report (YSR)		19 dropouts (24%) scored above the clinical treshold on the total problem score. 20% reported internalising problems and 30% externalising symptoms.<br><br>Logistic regression showed that girls scored significantly higher on internalising scores than males, weheras there was no gender difference for externalising problems.<br>Furthermore, girls reported significantly more thoughts of of suicide and self-harm than boys.	Self-report data<br>No confounding factors considered<br>Small sample size
Fergusson et Horwood<br>1998<br>Christchurch, New Zealand	Birth cohort, 21 waves<br><br>Christchurch Health and Development Study (CHDS)	N=969<br><br>0-18 years	Having left school without any qualifications by the age of 18<br>Having failed to obtain a grade a grade C or better in at least one School Certificate subject	Conduct problems at age 8 -> School dropout & early unemployment<br><br>With 2 hypotheses as intervening factors:<br><br>Conduct problems lead to impaired learning and educational achievement, which is translated into higher rates of dropout.<br>The effects of early conduct problems are mediated by adolescent behavioural patterns (substance use, peer affiliations, difficulties with school authority), that affect the student's commitment to educational achievement	Conduct problems<br><br>Cannabis use as intervening factor<br><br>Alcohol abuse as intervening factor	Rutter questionnaire<br>Conners questionnaire	Maternal age<br>Maternal education<br>Family SES<br>Family living standards<br>Child ethnicity<br>Family placement<br>Changes of residence<br>Changes of parents<br>Parental conflict<br>Childhood disadvantage<br>Maternal emotional responsiveness<br>Maternal avoidance of punishment<br>Gender<br>Intelligence (IQ) at age 8<br>Attentional problems at age 8	Children in the most disturbed 5% of the cohort had risks of leaving school without qualifications that were 1.8 times (CI=1.1-2.6) the risks of those in the least disturbed 50% of the cohort, and risks of unemployment were 2.2 times (CI=1.3-3.3) higher.Significant confounding factors included: early attentional problems, child IQ, maternal age, family SES, family living standards, parental change and parental conflict.<br><br>Even after control for potentially intevening educational variables, the association between early conduct disorder and school dropout remained significant.<br><br>Key mediating variables in this association were: peer affiliations ($p<0.0001$), cannabis use ($p<0.05$) and suspension from school ($p<0.05$).<br>There was no statistically detectable difference between boys and girls.	No significant attrition<br><br>Data collection from distinct sources
Rylance<br>1997<br>USA	longitudinal<br><br>National Longitudinal Transition Study of Special Education Students (NLTS)	N=664<br><br>18-27 years<br><br>77% male & 23% female<br><br>**Youth with severe emotional disturbances (SED)**	Non completion of HS	Risk/protective factors of HS dropout among youth with SED	SED		Gender<br>Age<br>Ethnicity<br>Parental education<br>Annual household income<br>Family composition<br>Mental health services use<br>Vocational education<br>Academic Competency	Nearly 50% of the youth with SED dropped out of HS (330/664).<br>The independent varibles predicted 35.7% of the overall variance of educational attainment.<br>A significant association with school dropout was found for parental income, age and competency.	Missing data<br><br>Lack of combined analyses
Schaufeli<br>1997<br>Netherlands	2 studies:<br>Study 1: college graduates, longitudinal, 5 waves<br>Study 2: School-leavers, longitudinal, 2 waves	Study 1: N=635<br>mean age: 22.8 years<br>63% male & 37% female<br><br>Study 2: N=767<br>mean age: 16.2 years<br>49% male & 51% female	Non completion of secondary school or a vocational training	unemployment -> mental health (causation hypothesis)<br>mental health -> employment (selection hypothesis)	Psychological distress	Symptom Checklist-90 (SCL-90), total score<br>General Health Questionnaire (GHQ-12)	Gender<br>Marital status<br>Type of education<br>Working in an unpaid job<br>Financial strain<br>Self-esteem<br>Locus of control<br>Attribution of unemployment<br>Problem-focused way of coping<br>Social support<br>Attitudes towards work and non-work	At time 0, when all intended dropouts were still at school, there was no significant difference in levels of mental health between the 2 groups.<br>At follow-up 1 year later, unemployment led to a deterioration in mental health ($t(13)=2.76$; $p<0.05$) whereas employment or continuing education improved mental health ($t(94)=3.12$ $p<0.01$ and $t(387)=2.62$ $p<0.01$ respectively) among early school leavers.<br>Unlike school dropouts, the unemployed college graduates did not suffer from poor mental health, thus the causation hypothesis was only supported for school dropouts. Among college graduates, neither the level of mental health, nor financial strain, nor personality characteristics seemed to play a role in finding a job.	The very small number (N=14) of dropouts who were unemployed did not allow to study determinants of employment among this group<br><br>Mental health was generally evaluated with a 12-item questionnaire which did not allow for any differenciation.
Oswald & Coutinho<br>1996<br>USA	cross-sectional	n.a.<br><br>>= 14 years<br><br>**Youth with severe emotional disturbances (SED)**	Non completion of school (un-defined)<br>1. graduation with diploma<br>2. graduation with certificate (other than regular diploma)<br>3. dropout<br>4. reaching maximum age	State-level child disability, demographic and economic factors -> School dropout	SED		Geographic region<br>Ethnicity<br>Rate of youth identified with SED<br>State per capita income<br>State per pupil revenue<br>Total expenditures per capita<br>Education expenditure per capita<br>Elementary and secondary education expenditure per capita	Less than one third of youth with SED graduated with a diploma and 38% graduated with a certificate.<br>In contrast, the rate at which students with SED dropped out was very high (40%), with important variations among states (9-76%). The dropout rate of students with SED was approx. double those for students with learning difficulties or mental retardation. Only 13% of the variance related to dropping out could be predicted by the demopgraphic, economic and disability variables.	Study of an extant database.<br><br>Lack of information on mediator variables.

**Reference**	**Study design**	**Sample**<br>**N, age range, gender**	**Dropout definition**<br>**HS: High school**<br>**GED: General Educational Development**	**Hypotheses**	**Disorder types**	**Evaluation of disorders**	**Confounding factors**	**Significant results**<br>**ORadj: adjusted odds ratio**<br>**CI: confidence interval**<br>**RR: relative risk**	**Limitations**
Franklin & Streeter<br>1995<br>Texas, US	cross-sectional	N=200<br><br>mean age: 17 years<br>55% male & 45% female<br><br>**middle class youth who dropped out of HS**	Noncompletion of HS	School, psychological and family correlates of HS dropout	Psychological/behavioral disorders (subscales of HAP)	Hilson Adolescent Profile (HAP)<br>Cassata History Questionnaire<br>Family Adaptability and Cohesion Test of Adult Basic Education (TABE)	School history<br>Demographic background<br>Academic skills<br>Family cohesion<br>Family adaptability<br>Family communication	The 3 most frequent reasons reported for dropout were inability to get along with the classroom environment, authority problems and truancy.<br>in the 9th grade, were underachievers and attended an average of 5 different schools.<br>46% of the dropouts fell in the clinical range of at least one of the HAP subscales, with drug use, alcohol use, law-society violations and anti-social risk taking as the most frequent risk behaviors.<br>79.8% of the school dropouts had a history of receiving counseling, mostly for family problems. Indeed, 74% of the sample viewed their families as having moderate to severe dysfunctioning.	Specific sample: only middle class youth who were willing to return to an alternative school<br>Self-report data<br><br>Descriptive statistics only
Kaplan et al. [4]<br>1994<br>Houston, US	longitudinal, 4 waves<br><br>comparison study:<br>group 1: HS dropouts<br>group2: HS graduates who did not enter college<br>group 3: normally enrolled students	N=4141<br><br>n.a.<br><br>45.5% male & 54.5% female	Noncompletion of HS<br><br>Not entering college	HS dropout -> Mental health problems in young adulthood	Global feelings of self-derogation<br>Anxiety<br>Cognitive distraction or disorientation<br>Depression<br>Coping ability<br>Sensitivity to opinions of others<br>Stability of self-feelings		Gender<br>Race<br>Father's educational level<br>Psychological functioning during junior HS	There was a significant association between early psychological dysfunctioning and HS dropout.<br><br>HS dropouts, compared to graduates but who did not enter college, were significantly more likely to show symptoms of psychological dysfunction in young adulthood, even after controlling for the prior level of psychological dysfunction.	Self-report data<br><br>Attrition bias
