# Supplementary material for: The downward spiral of mental disorders and educational attainment: a systematic review on early school leaving
Source: BMC Psychiatry. 2014 Aug 27;14:237. doi: 10.1186/s12888-014-0237-4 (PMC4244046; doi:10.1186/s12888-014-0237-4)
Supplement: Additional file 4: — Checklist summarising compliance with MOOSE guidelines, applied to Horwood et al., 2010. [file 12888_2014_237_MOESM4_ESM.pdf]

Additional file 4 - Checklist summarising compliance with MOOSE guidelines, applied to Horwood et al., 2010

**Reporting background should include**	
Problem definition	Yes
Hypothesis statement	Yes
Description	Yes
Type of exposure or intervention used	Yes
Type of study designs used	Yes
Study population	Yes
**Reporting methods should include**	
Description of relevance or appropriateness of studies assembled for assessing the hypothesis to be tested	Yes
Rationale for the selection and coding of data (eg, sound clinical principles or convenience)	Yes
Documentation of how data were classified and coded (eg, multiple raters, blinding, and interrater reliability)	No
Assessment of confounding (eg, comparability of cases and controls in studies where appropriate)	Yes
Assessment of study quality, including blinding of quality assessors; stratification or regression on possible predictors of study results	Yes
Assessment of heterogeneity	Yes
Description of statistical methods (eg, complete description of fixed or random effects models, justification of whether the chosen models account for predictors of study results, dose-response models, or cumulative meta-analysis) in sufficient detail to be replicated	Yes
Provision of appropriate tables and graphics	Yes
**Reporting of results should include**	
Graphic summarizing individual study estimates and overall estimate	Yes
Table giving descriptive information for each study included	Yes
Results of sensitivity testing (eg, subgroup analysis)	Yes
Indication of statistical uncertainty of findings	Yes
**Reporting of discussion should include**	
Quantitative assessment of bias (eg, publication bias)	NA
Justification for exclusion (eg, exclusion of non–English-language citations)	NA
Assessment of quality of included studies	Yes
**Reporting of conclusions should include**	
Consideration of alternative explanations for observed results	Yes
Generalization of the conclusions (ie, appropriate for the data presented and within the domain of the literature review)	Yes
Guidelines for future research	Yes
Disclosure of funding source	Yes

NA: not applicable

Overall risk bias: low
