# Supplementary material for: The downward spiral of mental disorders and educational attainment: a systematic review on early school leaving
Source: BMC Psychiatry. 2014 Aug 27;14:237. doi: 10.1186/s12888-014-0237-4 (PMC4244046; doi:10.1186/s12888-014-0237-4)
Supplement: Additional file 5: — Risk of bias for all included studies (n=51). [file 12888_2014_237_MOESM5_ESM.pdf]

Additional file 5 Risk of bias for all included studies (n=51)

For reference number please see reference list in manuscript.

− high risk of bias

+ low risk of bias

? risk of bias unclear

Mental disorders as a predictor of school dropout

(Studies investigating both sides of the mental health – dropout association are marked with *)

First author (reference)	Year	Selection bias	Detection bias	Attrition bias	Reporting bias	Confounding	Overall risk of bias (without considering confounding)	Comments
Hemphälä	2014	?	+	+	+	−	unclear	Small sample, no information about representativeness<br>Loss to follow-up: 18%, not differential<br>No confounding
Quiroga	2013	+	+	?	+	+	unclear	Particular sample of at-risk students, limited generalization<br>Non-response rate of 18%, no information about differences between respondents and nonrespondents<br>Control for sociodemographic and academic variables
Quiroga	2012	+	+	−	+	+	high	Particular sample of at-risk students, limited generalization<br>Differential loss to follow-up of 24.8% in 1 years: non-respondents were more likely to drop out<br>Control for sociodemographic and academic variables
McLeod	2012	+	+	?	+	+	unclear	No information about attrition, appendices are not available<br>Control for sociodemographic, family and academic variables

First author (reference)	Year	Selection bias	Detection bias	Attrition bias	Reporting bias	Confounding	Overall risk of bias	Comments
Borges	2011	+	+	+	?	+	low	Stratified multistage probability sample, representative<br>Response rate of 76.6%, weighting to avoid differential loss<br>No clear indication whether results present crude or adjusted odds ratios<br>Control for sociodemographic and family variables
Porche	2011	+	+	?	+	−	unclear	Low risk of recall bias despite efforts to use valid methods<br>No information about reponse rate<br>Control for psychiatric mobidity, service use and sociodemographic variables only
Kent	2011	+	?	−	+	−	high	Matching not taken into account in statistical analyses?<br>Loss to follow-up: 29.5% (364 participants out of 516)<br>Higher conduct disorder symptom rating for non-participants<br>Control for parental education and IQ
Breslau	2010	+	+	+	+	+	low	Low risk of recall bias despite efforts to use valid methods<br>Loss of 30% for longer term follow-up (> 1 year)<br>Control for sociodemographic and family variables (childhood adversities)
LeCook	2010	+	?	+	+	−	unclear	No information about considered disorder categories<br>Weighted response rates between 65.6% and 75.5%<br>Control for sociodemographic variables only
McCaffrey	2010	+	?	+	+	+	unclear	Unknown risk of detection bias regarding self-reported drug use given the discrepancies regarding dropout information (self-report versus school-based)<br>Follow-up response rates of 91%, 87% and 83%<br>Attrition handled by propensity score-based nonresponse weights, missing data imputation<br>Control for sociodemographic, academic and family variables

First author (reference)	Year	Selection bias	Detection bias	Attrition bias	Reporting bias	Confounding	Overall risk of bias	Comments
Horwood	2010	Meta-analysis evaluated according to MOOSE					low	Cf. additional file 5 for checklist of MOOSE criteria (Stroup et al. 2008)
Goulding	2010	?	+	?	?	−	unclear	Prevalence estimates would need an evaluation of sample representativeness<br>No information about response rate<br>Descriptive statistics only, no control for confounding
Marti	2010	+	+	−	+	−	high	Low risk of detection bias regarding self-reported drug use<br>Considerable loss to follow-up: 44%<br>No control for confounding
Legleye	2010	?	+	+	+	+	unclear	No information about inclusion/exclusion criteria<br>Low risk of detection bias regarding self-reported drug use<br>Response rate of 98%<br>Control for family and academic variables
Lee	2009	?	+	+	+	+	unclear	Weighted response rate of 70.8%, but potentially differential nonresponse regarding mental disorders<br>Low risk of detection bias regarding self-reported data<br>Control for sociodemographic and family variables (childhood adversities)
Fletcher	2008	?	+	+	+	+	unclear	No indication on inclusion/exclusion criteria, representativeness<br>Small but differential loss to follow-up (10% after imputation for mother education and family income), shown in appendix table<br>Control for sociodemographic, family and academic variables
Breslau	2008	+	+	+	+	+	low	Low risk of recall bias despite efforts to use valid methods<br>No control for co-occurring disorders, but cluster analyses<br>Response rate of 70.9%, non-respondents may be more likely to have psychiatric disorders<br>Control for sociodemographic and family variables

**First author (reference)**	**Year**	**Selection bias**	**Detection bias**	**Attrition bias**	**Reporting bias**	**Confounding**	**Overall risk of bias**	**Comments**
Degenhardt	2007	?	+	−	+	−	high	No indication on inclusion/exclusion criteria, representativeness<br>Low risk of detection bias regarding self-reported data<br>Differential loss to follow-up (25%): males and smokers were overrepresented in wave 8<br>Control for sociodemographic variables only
Bohon	2007	+	+	+	+	−	low	Blinding of interviewers, low risk of detection bias regarding self-reported data<br>Loss to follow-up: 19%, no differential attrition<br>Control for IQ, maternal education and adolescent behaviour problems
Barbaresi	2007	+	+	+	+	−	low	Inclusion/exclusion criteria well defined<br>Data from school and medical records<br>Outcome results based on case-control design<br>Control for matching variables (age, gender) only
Daniel *	2006	?	+	+	+	−	unclear	No information about representativeness, small sample<br>Small loss to follow-up: 7%<br>Control for sociodemographic variables and psychiatric morbidity
Kogan *	2005	+	+	+	+	+	low	Small but differential loss to follow-up (9%): non-participants were more likely to have dropped out, be male, smoke and have behavior problems<br>Control for sociodemographic and school-related variables
McShane	2004	+	+	−	+	+	high	Clinical sample<br>High and differential loss to follow-up (39%)<br>Control for sociodemographic, family and school-related variables
Fergusson *	2003	+	+	+	+	+	low	Loss to follow up (28-31%), weighting of data by the inverse of the probability of sample inclusion<br>Control for sociodemographic, academic and family variables

First author (reference)	Year	Selection bias	Detection bias	Attrition bias	Reporting bias	Confounding	Overall risk of bias	Comments
Ensminger	2003	+	?	–	+	–	high	Study population drawn from a specific community, dependent and confounding variables measured in 1966-1967<br>Maternal distress evlauation based on two simple questions<br>Differential loss to follow-up (23%): non-participants more likely to be poor and less likely to be living with both parents<br>Control for sociodemographic variables, mother's physical illness, child's social adjustment and psychological symptoms
Van Ameringen	2003	?	?	?	?	–	unclear	Small clinical sample with specific characteristics, exclusion criteria were unclear<br>Potential recall bias<br>No information about response rate<br>Descriptive statistics, unclear pourcentages (cumulative?)<br>Descriptive statistics, no control for confounding
Lynskey	2003	+	+	–	+	–	high	Low risk of detection bias regarding self-reported data<br>Differential loss to follow-up (21%): non-participants were more likely to be male, to have separated parents or parents with lower education<br>Control for sociodemographic, smoking and psychiatric morbidity
Van der Stoep	2003	?	+	?	?	–	unclear	No information about inclusion/exclusion criteria, non-response rate or representativeness (the title refers to US population while representativeness of the sample is compromised)<br>No information about attrition, control for socioeconomic status
Ellickson	2001	+	+	+	+	+	low	Self-reported smoking was cross-checked with saliva sample<br>Differential loss to follow-up (33%), but the sample was weighted to reduce bias by 90%<br>Control for sociodemographic and family variables

First author (reference)	Year	Selection bias	Detection bias	Attrition bias	Reporting bias	Confounding	Overall risk of bias	Comments
Bray	2000	?	+	−	+	+	high	Response rate and representativeness unclear<br>Low risk of detection bias regarding self-reported data<br>Loss to follow-up: 45%, compensated by a stratified random cross-sectional sample to be included for final analyses<br>Control for sociodemographic, family and academic variables
Hansen	1999	−	+	−	+	−	high	Variables measured in mid until late 80s, very small sample (n=36)<br>Differential loss to follow-up (44%) for ADHD group<br>Descriptive statistics, no control for potential confounding
Miech *	1999	+	+	+	+	+	low	Birth cohort, 9 waves<br>Categorical and dimensional assessment of mental disorders<br>Loss to follow-up: 9%<br>Control for sociodemographic and academic variables
Krohn *	1997	+	+	+	+	+	low	Acceptable loss to follow-up (22%)<br>Control for sociodemographic, family and academic variables
Kessler	1995	+	+	+	+	+	low	Low risk of recall bias despite efforts to use valid methods<br>Response rate of 82.4%, non-respondents may be more likely to have psychiatric disorders<br>Control for sociodemographic and academic variables

**School dropout as a predictor of mental disorders**								
**First author (reference)**	**Year**	**Selection bias**	**Detection bias**	**Attrition bias**	**Reporting bias**	**Confounding**	**Overall risk of bias**	**Comments**
Apantaku-Olajide	2014	?	+	?	+	−	unclear	No information about representativeness of the sample<br>No information about differential attrition<br>Descriptive statistics, no control for potential confounding
Ohayon	2014	+	+	?	+	?	unclear	No information about response rate<br>Univariate logistic regressions?
Benjet	2012	+	+	+	+	−	low	Sample designed to be representative of Mexico City youth<br>Response rate of 71%, weighting of data to account for differential loss<br>Control for sociodemographic variables only
Lee	2010	+	+	?	+	−	unclear	Unclear information about loss to follow-up<br>Control for psychiatric morbidity and family variables (8 risk factors were tested indpendently and simultaneously)
Benjet	2009	+	+	?	+	+	unclear	Unclear information about response rate: were non-participnats different from participants?<br>Control for adolescent burden, sociodemographic and family variables
Crosnoe	2007	+	+	+	+	+	low	Acceptable overall loss to follow-up: 23%<br>Differential attrition across waves and unequal probability of selection into the final sample were balanced by weighting<br>Control for sociodemographic, family and school-related variables

First author (reference)	Year	Selection bias	Detection bias	Attrition bias	Reporting bias	Confounding	Overall risk of bias	Comments
Fothergill	2007	?	+	+	+	+	unclear	Study population drawn from a specific community, independent and confounding variables measured in 1966-1967<br>Acceptable but differential loss to follow-up (23%), however balanced by multiple imputation<br>Control for gender and mother's drug use
Williams	2006	+	+	?	+	+	unclear	Differential nonresponse was compensated by weighting, however no detail provided<br>Control for sociodemographic and academic variables
Harford	2006	+	+	?	+	−	unclear	Low nonresponse rate, but no information about differences between respondents and nonrespondents<br>Control for sociodemographic and behavioral characteristics
D'Amico	2005	+	+	+	+	+	low	Substantial sample attrition over the 11-year period (70%), accounted for by weighting techniques that reduced attrition bias by 90%<br>Control for sociodemographic, family and deviance variables
Liem	2001	?	+	?	+	+	unclear	Unclear information about inclusion/exclusion criteria<br>No information about attrition<br>Control for sociodemographic and family variables
Obot	2000	?	+	?	+	−	unclear	5 matched controls per case? Unclear information<br>Low risk of detection bias regarding self-reported data<br>No information about response rate<br>Control for sociodemographic variables only
Obot	1999	?	+	?	+	−	unclear	No indication about the number of matched controls per case<br>Low risk of detection bias regarding self-reported data<br>No information about response rate<br>Control for sociodemographic variables only

**First author (reference)**	**Year**	**Selection bias**	**Detection bias**	**Attrition bias**	**Reporting bias**	**Confounding**	**Overall risk of bias**	**Comments**
Obot	1999b	?	+	?	+	−	unclear	No indication about the number of matched controls per case<br>Low risk of detection bias regarding self-reported data<br>No information about response rate<br>Control for sociodemographic variables only
Crum	1998	+	+	−	+	−	high	Study population drawn from a specific community, independent and confounding variables measured in 1966-1967<br>Acceptable but differential loss to follow-up (23%): non-participants more likely to be poor and to have dropped out of school<br>Control for gender only
Gfoerer	1997	?	+	?	+	−	unclear	Unclear information about inclusion/exclusion criteria, gender<br>Low risk of detection bias regarding self-reported data<br>Response rate of 83%, no information about differences between participants and non-participants<br>Control for sociodemographic variables only
Tresidder	1997	?	+	−	+	−	high	Representativeness unclear, but difficult to estimate cinsidering the target population<br>Low risk of detection bias regarding self-reported data<br>Very low response rate: 17%<br>Control for gender and method of recruitment respectively
